# Comparing the Variants of Iron Oxide Nanoparticle-Mediated Delivery of miRNA34a for Efficiency in Silencing of PD-L1 Genes in Cancer Cells

**DOI:** 10.3390/pharmaceutics15010215

**Published:** 2023-01-08

**Authors:** Richa Pandey, Feng-Shuo Yang, Vyshnav Punnath Sivasankaran, Yu-Lun Lo, Yi-Ting Wu, Chia-Yu Chang, Chien-Chih Chiu, Zi-Xian Liao, Li-Fang Wang

**Affiliations:** 1 Department of Medicinal and Applied Chemistry, Kaohsiung Medical University, Kaohsiung 80708, Taiwan; 2 Department of Laboratory Medicine, Kaohsiung Medical University Hospital, Kaohsiung 80708, Taiwan; 3 SRM Institute of Science and Technology, R2FV+6Q7, Potheri, SRM Nagar, Kattankulathur 603203, Tamil Nadu, India; 4 Department of Biotechnology, Kaohsiung Medical University, Kaohsiung 80708, Taiwan; 5 Department of Medical Research, Kaohsiung Medical University Hospital, Kaohsiung 80708, Taiwan; 6 Institute of Medical Science and Technology, National Sun Yat-sen University, Kaohsiung 80424, Taiwan

**Keywords:** iron oxide nanoparticles, transfection efficiency, PD-L1 gene, miRNA34a, immunotherapy

## Abstract

The blocking of programmed death-ligand 1 (PD-L1) in tumor cells represents a powerful strategy in cancer immunotherapy. Using viral vectors to deliver the cargo for inactivating the PD-L1 gene could be associated with host cell genotoxicity and concomitant immune attack. To develop an alternative safe gene delivery method, we designed a unique combination for miRNA34a delivery using a transgene carrier in the form of iron oxide magnetic nanoparticles (IONPs) via magnetofection to downregulate PD-L1 expression in cancer cells. We synthesized IONPs of multiple shapes (IONRs (iron oxide nanorods), IONSs (iron oxide nanospheres), and ITOHs (iron oxide truncated octahedrons)), surface-functionalized with polyethyleneimine (PEI) using the ligand exchange method, as gene delivery systems. Under the guidance of an external magnetic field, PEI@IONPs loaded with plasmid DNA (DNA/PEI@IONPs) encoding GFP showed high transfection efficiency at different weight ratios and time points in A549 and MDA-MB-231 cells. Additionally, the DNA/PEI@IONPs with miRNA34a inserts under a static magnetic field resulted in significant knockdown of the PD-L1 gene, as demonstrated via immunoblotting of the PD-L1 protein. Among the three shapes of IONPs, IONRs showed the highest PD-L1 knockdown efficiency. The genetic expression of miRNA34a was also studied using qPCR and it showed high expression of miRNA in cells treated with PEI@IONRs. Flow cytometry and a live/dead assay confirmed apoptosis after transfection with miRNA34a. To conclude, in this paper, a promising transgene carrier with low cost, negligible cytotoxicity, and high transfection efficiency has been successfully established for miRNA gene delivery in the context of cancer immunotherapy.

## 1. Introduction

Conventional techniques for the delivery of therapeutic payloads are inefficient in altering the metabolic or genetic circuits inside cells due to their inability to perfectly locate their target [1]. Imprecise interaction of genes/drugs with their target leads to many catastrophic consequences such as unwanted toxicity or the alternation of specific gene expression [2]. To harmonize the controlled release of drugs, a plethora of vehicles have been engineered for the synaptic delivery of genes/drugs inside the cells, to enhance therapeutic efficacy [3]. The advancement of nanotechnology has brought about a paradigm shift in nanomedicine and remains the hallmark of biomedical research for exploring the fundamental properties of nanomaterials used in nanomedicine [4]. Altering the shape, size, and surface area of nanoparticles changes their optical, magnetic, electronic, and biological attributes. The intricate relationship between the physical properties of nanomaterials and the interaction of genes is not well comprehended, and the efficiency of the treatment and diagnosis of illness is still in its infancy [5]. Biological macromolecules such as DNA (2 nm) and cellular membranes (10 nm) lie within a nanometric scale [5,6], and owing to this property, the surface and shape of nanomaterials can easily be tuned to optimize the interaction with the target. Such important modulations facilitate the response to external stimuli and favorable interactions with specific cellular markers in a pre-controlled manner to launch efficient pharmacological responses with minimal side effects [7].

There is an array of metallic and polymeric nanoparticles used for gene delivery, such as polyethyleneimine-orchestrated chitosan oligosaccharide (CSO-PEI) with hyaluronic acid and small interfering RNA, dextran-coated nanoparticles, gold nanoparticles, graphene–iron oxide composites, and iron oxide nanoparticles [8,9,10]. These nanomaterials work in response to an external stimulus such as pH, temperature, pressure, electricity, and magnetic field [9]. Low transfection efficiency of non-metallic gene carriers, such as adenovirus and other polymeric vehicles, restrict their utility in gene therapy [11]. To overcome these restraints, magnetofection (gene delivery under magnetic influence) is an efficient technique to improve transfection efficiency [12]. Magnetic targeting exploits superparamagnetic nanoparticles such as iron oxide nanoparticles (IONPs) to accumulate genes in the tissues using the local magnetic field [13], where the leaching of iron ions will not reach beyond toxic limits owing to the presence of various molecular systems that remove them from the body [14]. However, to deplete the toxicity of IONPs, the surfaces of nanoparticles with various surface orchestration moieties have been fabricated for drug delivery [15].

In this study, we fabricated various shapes of IONPs such as spheres, octahedrons, and nanorods as efficient vehicles for the delivery of microRNA34a (miRNA34a) for silencing the programmed death-ligand 1 (PD-L1)gene in cancer cells. PD-L1 is a glycoprotein expressed on the cell surface membrane, which activates programmed cell death protein 1 (PD-1) receptors located on the T cell surface and leads to the inhibition of T cell activity [16]. PD-L1 plays many positive roles in the immune system such as mediating immune tolerance and protecting tissues from immune system attack, but PD-L1/PD-1 interaction plays a negative mediator role in responses of the immune system [16]. The tumor microenvironment is composed of cells that express the death-ligand 1 protein on cell surfaces, which binds to its cognate receptor PD-1, which is present on activated T cells; this immunological interaction often results in the functional impairment of infiltrated T cells and is one of the survival strategies adopted by cancer cells to escape immune attack [17].

Currently, to provide additional synergy for improving the outcome of blocking PD-L1/PD-1 interaction, the Shen’s group utilized IR-LND- or MHI148-modified bovine serum albumin in conjunction with the multi-kinase inhibitor sorafenib to show significantly increased oxygen perfusion in tumor cells. Tumor re-oxygenation suppresses the hypoxic environment and has been shown to enhance its killing through cytotoxic T cells [18,19]. However, most immunotherapy approaches that target cancer cells involving the usage of oncogenic viruses or non-specific drugs result in unwanted side effects. To address these shortcomings, we have developed non-toxic iron oxide nanoparticles conjugated with miRNA that target the checkpoint inhibitor as a safer and more potent immunotherapy approach to killing tumor cells. MicroRNAs (miRNAs) are usually ~22 nt nucleotides and are often considered small non-coding RNAs. They have been strongly implicated in tumor suppression and oncogenic processes [20] and are capable of gene knockdown via the decay or inhibition of mRNA translation [16,21]. Our approach relies on targeting PD-L1 in tumor cells via the miRNA34a that regulates the expression of the checkpoint inhibitor. Several researchers have utilized the miRNA34a gene for targeting PD-L1 in a new immunotherapy method for the treatment of various cancer diseases [22,23,24,25].

**Scheme 1:**
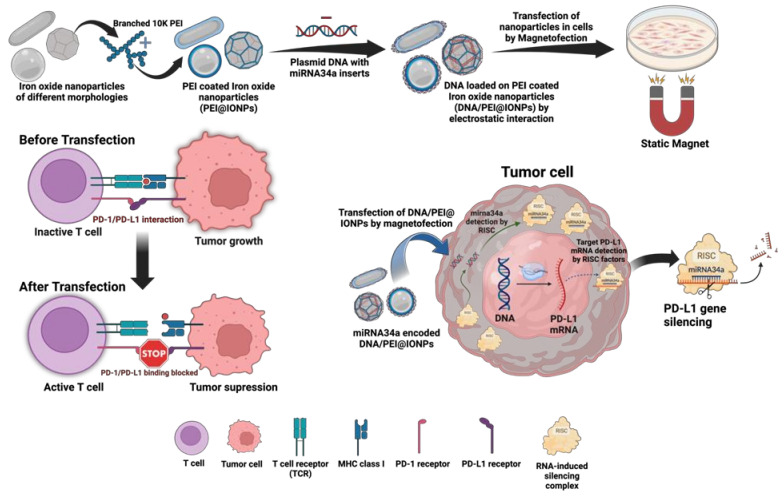
Illustration of the synthesis of iron oxide nanoparticles (IONPs) with three different morphologies, and surface functionalization with positively charged branched polyethylenimine (B-PEI). Plasmid DNA (pDNA) with miRNA34a inserts is encapsulated on the surface of B-PEI-coated IONPs (PEI@IONPs) with electrostatic interactions as DNA is negatively charged. The PEI@IONPs and pDNA magnetoplexes are transfected into cancer cells using a static external magnet to study gene delivery efficiency. Upon PD-L1 interaction with the programmed cell death protein 1 (PD-1) on T cell surfaces, the T cells become inactive and unable to help the immune system to fight against tumor growth. The PD-L1 present on the tumor cell surfaces is targeted by miRNA34a-encoded pDNA using PEI@IONPs. pDNA internalization and release inside the cell happen mostly via the enhanced permeability and retention (EPR)/proton sponge effects, and the desired PD-L1 gene silencing takes place gradually due to various factors inside the cell.

The combined efforts to comprehend the role of different shapes and sizes of IONPs have unlocked the importance of shape dependency in gene delivery vehicles. The different shapes of IONPs have helped us to develop a better understanding of the impact of their shapes and sizes for gene delivery applications, and coating them with PEI has enhanced their functionality, thereby making them accessible in the field of target-specific drug/gene delivery for cancer treatment [26]. Owing to the general lack of reports on comprehending the impact of the shapes and sizes of IONPs on their efficiency as vehicles to deliver the miRNA34a gene for cancer treatment, we aimed to target the PD-L1 gene expressed in triple-negative breast cancer (TNBC) and non-small-cell lung cancer (NSCLC) cells with miRNA34a pDNA using different shapes of IONPs while checking the efficiency of each nanoparticle with respect to its shape and size. The PEI coating enhances nanoparticle uptake into cells, which helps in facilitating endosomal escape for the pDNA delivery, as well as facilitating miRNA delivery [27]. Our goal is to compare and determine efficiency among different shapes of IONPs, and to discover which can deliver the maximum amount of miRNA34a to inhibit PD-L1 expression in cancer cell lines. This study unlocks the potential of shape- and size-specific IONPs under magnetic influence to enhance their transfection efficacy as a PD-L1-silencing agent using miRNA34a (Scheme 1).

## 2. Materials and Methods

### 2.1. Synthesis of Different Shapes of Iron Oxide (Fe_3_O_4_) Nanoparticles

#### 2.1.1. Iron Oxide Nanorods

The synthesis of iron oxide nanorods (IONRs) was performed using a two-step method. In the first step, rod-shaped ß-FeOOH was synthesized as a template using a modified method reported in the previous paper [27]. Briefly, rod-shaped iron(III) oxide-hydroxide or ferric oxyhydroxide (FeOOH) nanostructures of approximately 70 nm in length were obtained via hydrolysis in an aqueous solution of iron trichloride hexahydrate (FeCl_3_.6H_2_O) (Showa Chemical Co. Ltd, Akasaka, Minato-ku, Tokyo, Japan) mixed with deionized (dd) water (BioLab, Sartorius, Arium® Pro, Barcelona, Spain) and 0.2 mL linear polyethyleneimine (PEI) (Sigma-Aldrich, St. Louis, MO, USA). This reaction mixture was heated at 80 °C under magnetic stirring for 2 h to obtain a uniform structure of FeOOH with a rod shape. In the second step, 3 mL of ß-FeOOH with 10 mL tetraethylene glycol (TEG) (ACROS Organics, Thermofisher, MA, USA) and 1g of branched PEI 10K (B-PEI, Sigma-Aldrich) were put into a two-neck round-bottom flask. Under magnetic stirring, the above solution was heated to 80°C for 10 min. To remove water, ethanol, and other contaminants, the temperature was increased to 120 °C for 30 min under vacuum, and the temperature was raised to 230 °C (4 °C/min) for 1 h for further reducing. A black-colored product was obtained after completion of the reaction, which was further used for experiments after washing with ethanol/acetone several times and storage in ethanol.

#### 2.1.2. Iron Oxide Nanospheres

Iron oxide nanospheres were synthesized using a standard protocol [28]. The synthesis method was comprised of two critical steps. In the first step, an iron oleate complex was synthesized by adding sodium oleate (Sigma Aldrich) (147.05g, 483 mmol) and 500 µL hexane (Seedchem, VIC, Melbourne, Australia) to a three-neck round-bottom flask under constant stirring. To the above mixture, 300 mL ethanol was added and heated to 40 °C under constant stirring. To initiate the formation of iron oxide nanospheres, iron(III) chloride solution (trichloride hexahydrate 43.518g; 161 mmol in 100 mL DD water under stirring for 30 min) was added to the reaction vessel and the mixture was refluxed to 60 °C after purging with argon for 1 min. After refluxing under stirring for 4 h, the solution was cooled to 50 °C; the bottom layer was discarded and the red-colored liquid was collected in an Erlenmeyer flask containing 50g sodium sulfate. The solution was swirled occasionally for 10 min using a rotary evaporator, and then, the solution was filtered [29]. In the second step, the iron oleate complex and oleic acid were dissolved in 200g of 1-octadecene at room temperature. The reaction mixture was heated up to 320 °C at a heating rate of 3.3 °C min^−1^ for 30 min. After reaching at 320 °C, an intense reaction occurred, leading to the transparent solution turning turbid and brownish black. The resulting solution was cooled down to room temperature and precipitated by adding 500 µL of ethanol, and the product was obtained via centrifugation.

#### 2.1.3. Iron Oxide Truncated Octahedron

The synthesis of the truncated octahedron was conducted using the protocol reported in [30]. Briefly, magnetite nanoparticles were prepared using a thermal decomposition reaction in which approximately 22 nm sized truncated octahedron Fe_3_O_4_ nanoparticles were synthesized using 1.42g of tris(acetylacetonato) iron(III) {Fe(acac)_3_} (ACROS Organics), 0.57 mL of oleic acid, and 20 mL of trioctylamine. The solution was then refluxed at 310 °C in an Ar environment for 30 min. The final product was cooled to room temperature and the precipitate was collected and washed using a toluene/ethanol (*v/v* = 1:4) solution.

#### 2.1.4. Surface Functionalization of Fe_3_O_4_ Nanoparticles (Nanorods, Nanospheres, and Truncated Octahedrons) by B-PEI 10K

Iron oxide nanoparticles (IONPs) were subjected to surface orchestration using a ligand exchange method with branched PEI 10K. In a two-neck round-bottom flask, 3 mL of the IONPs of different shapes were mixed with 2 mL of PEI, along with the addition of 5 mL DD water. The following mixture was heated up to 80 °C under constant magnetic stirring and an inert environment using Ar gas for 8 h [27]. The mixture was cooled down and washed several times in acetone/ethanol (*v/v* = 1:1) solution. The final product was suspended in DD water for further use.

### 2.2. Characterizations

Morphologies were characterized using a transmission electron microscope (TEM, Jeol TEM-1200; Tokyo, Japan). The magnetite characteristics were studied using a powdered X-ray diffractometer (XRD) (Shimadzu Cu Kα radiation at λ=1.54060 Å, Tokyo, Japan). The size and surface charge of the PEI-modified nanoparticles were confirmed using a dynamic light scattering devise (DLS, ELSZ-2000, Otsuka Electronics, Osaka, Japan). The surface chemistry of the nanoparticles was determined using Fourier transform infrared spectroscopy (FTIR, Bruker ALPHA-T spectrometer, Billerica, MA, USA). In addition, the stability of the nanoparticles was studied using the Zetasizer (ELSZ-2000, Otsuka Electronics) to determine the change in size upon storage at 4 °C for at least two weeks. The magnetic power of the nanoparticles was measured using a Superconducting Quantum Interference Device (SQUID, Quantum Design MPMS, Darmstadt, Germany). The temperature dependence of the magnetic susceptibility (ø) M/H was measured as a function of temperature in a magnetic field, where M is the magnetization and H is the applied magnetic field.

### 2.3. Synthesis of Magnetoplexes and Transfection Efficiency Studies

Magnetoplexes were fabricated using the different concentrations of iron oxide nanoparticles, i.e., PEI-coated nanorods, nanospheres, and truncated octahedrons with GFP-tagged pDNA. The ratio of NP:pDNA was optimized in an aqueous medium for increased transfection efficiency. The ideal weight ratio of the nanoparticles and pDNA for maximum transfection efficacy was set to 5 (*W/W* = 5). The magnetoplexes were suspended in a serum-free medium (total volume 500 μl). The cells were cultured (7 × 10^4^/well) in a 12-well plate and incubated for 24 h. The magnetoplexes suspended in serum-free medium were added to the medium containing cells and incubated for 45 min on a magnetic plate. To comprehend the impact of a magnetic field on the transfection efficacy, the study was performed in the presence and absence of a static magnetic array (Chemicell GmbH, Berlin, Germany). After 45 min of incubation, the medium was replaced with 1 mL of fresh medium with 10% FBS, and the cells were incubated for another 48 h. The efficacy of the transfection was assayed using fluorescence microscopy and flow cytometry.

### 2.4. Cell Culture and Cytotoxic Studies

A549 (non-small-cell lung carcinoma/NSCLC) and MDA-MB-231 (triple-negative breast cancer/TNBC) cells were cultured in Dulbecco’s Modified Eagle Medium (DMEM) and Minimum Essential Medium (MEM) supplemented with 10% fetal bovine serum (FBS) and 100 μg/mL penicillin–streptomycin, at 37 °C and 5% CO_2_. The medium was replaced with a fresh one after 3 days, and the cells were sub-cultured until 95% confluency. The cytotoxicity of magnetic nanoparticles was determined using the MTT assay. A549 and MDA-MB-231 cells were seeded in 96-well culture plates (the density of the cells was 5 × 10^3^/well). The cytotoxicity was measured at different concentrations (0–200 µg/mL) after incubation with the PEI@IONPs for 48 h at 37 °C (*n* = 24). Additionally, the cytotoxicity was also measured after the transfection of PEI@IONPs with plasmid DNA, and the results were compared with PEI 25K and PolyMag, respectively (*n* = 3). For this study, A549 and MDA-MB-231 cells were seeded in 12-well culture plates (the density of the cells was 7 × 10^4^/well). The PEI@IONP-to-pDNA ratio (NP:pDNA) was fixed at a weight ratio of 5 (*W*/*W* = 5) and incubated under a static magnet array for 45 min, and the MTT assay was performed after 48 h of incubation.

### 2.5. Western Blot Analysis

MiRNA34a-transfected A549 and MDA-MB-231 cells were cultivated and lysed using a protein lysis buffer. The protein concentration of the cell lysate was determined using a BCA protein assay kit (Pierce ®, Thermo Scientific, Rockford, IL, USA). A fixed concentration of protein (40 μg) was separated using a 10% SDS-PAGE gel and transferred to a Poly(vinylidene fluoride) (PVDF) membrane. The membrane was blocked with a 5% skim milk powder in a phosphate-buffered saline solution with 0.1% Tween™ 20 (PBST) at room temperature for 1 h; later, the membrane was incubated with the required primary antibody (PD-L1, AKT, pAKT, PTEN, Caspase 3, cleaved caspase 3, and Caspase 7) and ß-tubulin as a control, at 4 °C overnight. After incubation, the membrane was washed with PBST for 10 min three times, and then, incubated with a secondary antibody for 1 h. Following the incubation, the membrane was washed with PBST four times for 10 min, visualized using enhanced chemiluminescence, and then, detected using an auto-imaging system (Amersham Imager 680, GE Healthcare and Bioscience ab, Princeton, NJ, USA).

### 2.6. Live/Dead Assay to Analyze Apoptosis via Cell Staining

A549 and MDA-MB-231 cells (7 × 10^4^ cells/well) were seeded into 12-well plates and incubated at 37 °C overnight; later, they were transfected with miRNA34a (3μg) using PEI@IONPs at a weight ratio of 5 (*W*/*W* = 5) and commercially available PEI and PolyMag, for 45 min under a magnetic array in a serum-free medium. The cells with the test samples were incubated in substituted DMEM containing 10% FBS for another 48 h. After incubation, the cells were washed twice with 1X PBS and stained with calcein-AM (Sigma Aldrich) and propidium iodide (PI) (Sigma Aldrich) (according to the protocol given by Cellink accessed on 1 June 2022 onwards (https://www.cellink.com/wp-content/uploads//2019/03/Viability-Protocol-Calcein-AM-PI_07-Mar-2019.pdf).

### 2.7. Flow Cytometry for Cell Apoptosis

A549 and MDA-MB-231 cells were plated at a density of 1 × 10^5^ cells/well in 6-well plates for the apoptosis assay. The cells were transfected with miRNA34a (3μg) using PEI@IONPs at a weight ratio of 5 (*W/W* = 5) and commercially available PEI and PolyMag, for 45 min under a magnetic array in a serum-free medium, and incubated in substituted DMEM containing 10% FBS for another 48 h. The cells were collected after 48 h of incubation and analyzed using an Annexin V-FITC apoptosis detection kit according to the manufacture’s protocol. Briefly, the cells were washed with 1X PBS and trypsinized for collection, and the cell pellets were collected via centrifugation at 1500× *g* rpm for 5 min. The cell pellets were incubated with Annexin V-FITC/PI solution at room temperature for 30 min and assayed using a flow cytometer (Beckman Coulter) and WinMDI software.

### 2.8. Quantitative Real-Time Polymerase Chain Reaction (qRT-PCR)

A549 and MDA-MB-231 cells were subjected to gene expression studies to confirm the presence of miRNA34a post-transfection. Total RNA was extracted from A549 and MDA-MB-231 separately using a 3-Zol (Trizol) reagent according to the standard protocol. The ratio of A260/A280 was measured using a nanodrop (Thermo Scientific™), and it came out between 1.8 and 2, indicating RNA purity. Total RNA was reverse-transcribed to complementary DNA (cDNA) using a universal method with a reverse transcriptase enzyme via incubation for 1 h at 37 °C, and then, terminated at 85 °C for 5 min to inactivate the enzyme in the PCR machine (Takara Bio USA, Inc., San Jose, CA, USA). Further, cDNA was used as a template and U6 RNA as the control gene, and the expression levels of miRNA34a were assayed using a SYBR green qRT-PCR universal method. Quantitative real-time polymerase chain reaction (qRT-PCR) was performed using a SYBR Green PCR Master Mix system (Applied Biosystems, cat. 4309155); the forward and reverse primers, the temperature, and the time conditions of the reaction are as follows: 95 °C for 10 min to activate the reaction; 40 continuous cycles of 95 °C for 15 s and cooling to 60 °C for 1 min; and 95 °C for 15 s, cooling to 60 °C for 1 min, and warming to 95 °C for 15 s to measure the cycle threshold (Ct). The fold change (FC) difference in mRNA expression between the experimental group and the control group was calculated using the 2^−^△△^Ct^ equation. The results were analyzed using StepOne Software v2.2.2 (Applied Biosystems, Foster, CA, USA) [31].

The primer sequences used for miRNA34a: hsa-miR-34a-5p were [32]:

**sense, 5’-GCAGTGGCAGTGTCTTAG-3’**

**antisense, 5’-GGTCCAGTTTTTTTTTTTTTTTACAAC-3’**

### 2.9. Statistical Analysis

The means and standard deviations (SD) of the data were calculated. Differences between the groups were tested using a Student’s t-test, and *p* * < 0.05 and *p* ** < 0.01 were considered to be significant.

## 3. Results and Discussion

### 3.1. Synthesis and Characterizations of Iron Oxide Nanoparticles with Different Shapes and Sizes

**Figure 1:**
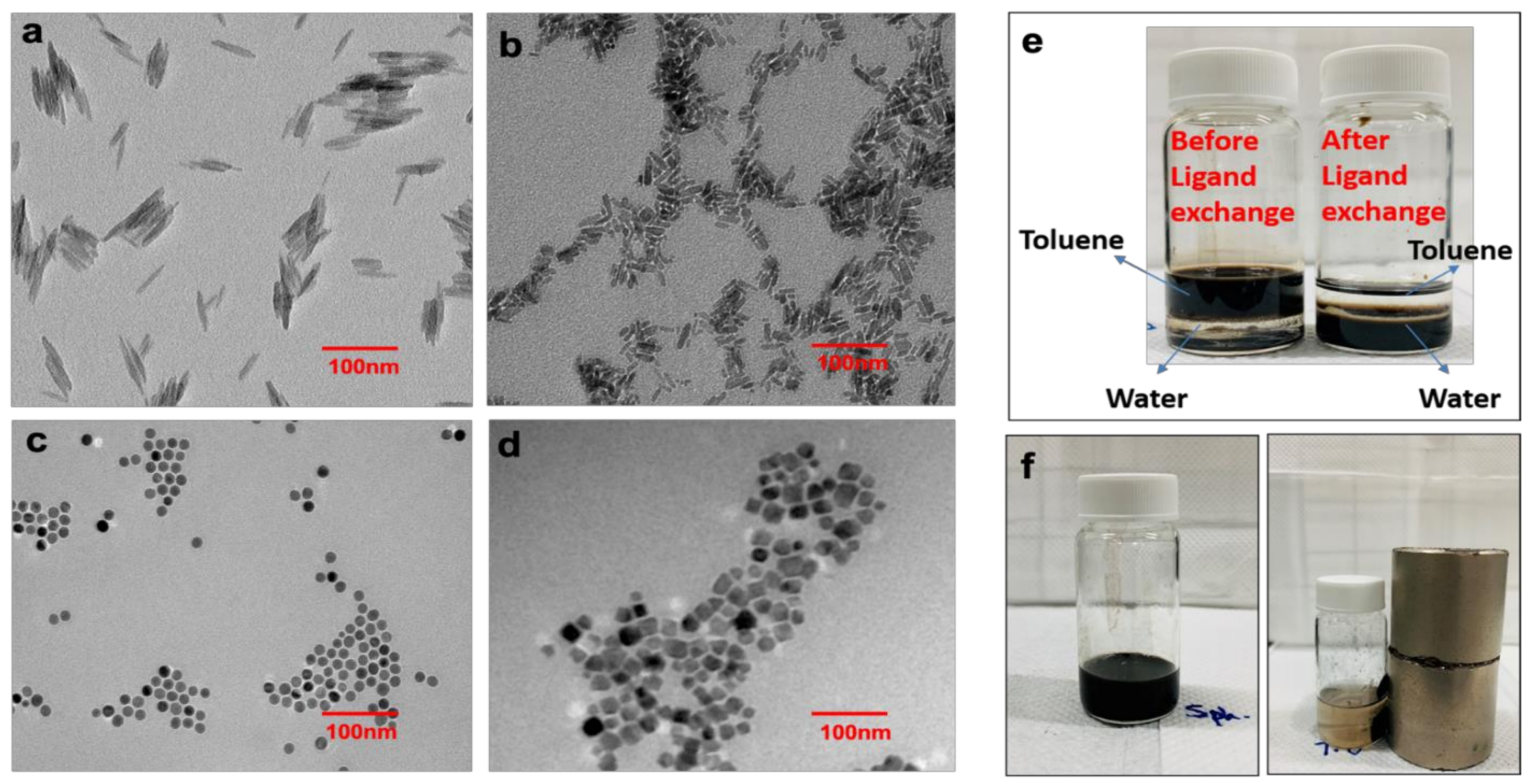
**TEM images of iron oxide nanoparticles displaying monodispersed and diverse morphologies.** (**a**) FeOOH nanorod precursors after hydrolysis of FeCl_3_; (**b**) Fe_3_O_4_ nanorods after reduction of FeOOH using branched PEI (B-PEI) and tetraethylene glycol (TEG) at 230 °C; (**c**) Fe_3_O_4_ nanospheres formed via thermal decomposition of metal-oleate precursors at 320 °C; (**d**) Fe_3_O_4_ truncated octahedrons formed via thermal decomposition reaction of Fe(acac)_3_, oleic acid, and trioctylamine (Scale bar: 100 nm); (**e**) the properties of nanoparticles before and after the surface functionalization using B-PEI 10K via a ligand exchange method; and (**f**) demonstration of the magnetic nature of the Fe_3_O_4_ nanoparticles using a strong static magnet.

The strategy for the synthesis of iron oxide nanorods (IONRs) with different shapes and sizes is given in the Supplementary Materials (Figure S1). The crucial parameters for tuning up the shapes and sizes of the IONRs were temperature and time, the presence amount of polyethyleneimine (PEI) and tetraethylene glycol (TEG). For the fabrication of IONRs, the precursor iron(III) oxide-hydroxide or ferric oxyhydroxide (FeOOH) was synthesized using a published protocol without any functionalization [27]. TEM images display different morphologies of Fe3O4 under different temperatures and surface modification (Figure 1). Spindle-shaped β-FeOOH nanorods (NR) have a larger morphology with an approximate length of 70 nm (Figure 1a). The transformation (FeOOH to Fe3O4) phase can be seen in Figure 1b, where FeOOH NRs are reduced to the Fe3O4 phase using TEG and 10,000-molecular-weight (10K MW) linear PEI at 230 °C, retaining the shape and size of the NRs. The transformation probably occurs due to the solvent and reductant properties of TEG [33,34]. The addition of the appropriate amount of PEI to the reaction mixture leads to the formation of FeOOH NRs with a thinner diameter which, after further reduction, enhances the morphology of Fe_3_O_4_ nanoparticles [27].

Figure 1c shows the formation of nanospheres whose uniform and monodispersed shapes have a diameter of approximately 12 nm. The iron oxide nanospheres were prepared using the published protocol for the thermal decomposition of Fe(oleate)_3_ at 320 °C for 30 min. The possible mechanism reported in previous studies is nucleation that occurs at 200–240 °C, triggered by the dissociation of one oleate ligand from the Fe(oleate)_3_ precursor due to CO_2_ elimination [28]. The major growth occurs at ~300 °C, initiated by the dissociation of the remaining two oleate ligands from the iron oleate species, although a slow growth rate seems to occur at < 250 °C. The monodisperse nanospheres were successfully synthesized due to the different temperature dependence of nucleation and growth kinetics. Figure 1d shows the truncated octahedrons prepared according to a published method using the thermal decomposition reaction of iron acetylacetonate [Fe(acac)_3_], oleic acid, and trioctylamine [30]. The formation process of the octahedral structure was studied in earlier experiments by monitoring the reaction solution at 240, 300, 360, and 380 °C, respectively [35]. When the nanoparticles approached the decomposition temperature of iron(III) oleate, they were several nanometers in size with no regular shape. The authors concluded that the nuclei formed at this stage would affect the final product’s size dispersion and shape significantly; moreover, especially at 300 °C, the nanoparticles would evolve up to 10 nm, forming a truncated octahedron shape [35].

The nanoparticles were further surface-functionalized with 10,000-branched PEI (B-PEI 10K) using a ligand exchange method. The surface coating method was referenced from a published protocol [27]. After the ligand exchange with PEI, the surface properties of the nanoparticles changed from hydrophobic to hydrophilic, which confirms that the PEI was successfully coated on the surface of the nanoparticles (Figure 1e). The surface modification of IONPs with B-PEI 10K at 80 °C showed no significant changes in the size and shape of the nanoparticles (Figure S2). The PEI coating has the potential to deliver pDNA and miRNA, which further enhances the capacity of the nanoparticles to enter the cells and deliver the desired nucleotide via the endosomal escape or the proton sponge effect mechanisms [27,36].

To confirm the superparamagnetic property of the Fe_3_O_4_ nanoparticles, a static magnet was placed near the nanoparticles. Figure 1f shows that the nanoparticles are attracted by the static magnet (left) but, in the absence of the static magnet, the nanoparticles return to their solution form (right). This result indicates the superparamagnetic property of the nanoparticles. Here, we successfully synthesized three kinds of superparamagnetic PEI-coated monodispersed Fe_3_O_4_ nanoparticles.

The hydrodynamic sizes and zeta potentials of nanoparticles with different morphologies before and after PEI coating are given in the Supplementary Materials (Table S1 and Table S2). The hydrodynamic size of PEI@IONPs was found to be in a desirable range for maximum transfection efficacy. The addition of an appropriate amount of PEI to the reaction resulted in the formation of thinner-diameter nanoparticles with good stability [27]. A stability test of PEI@INOPs was also carried out after DNA binding, and their surface charge was analyzed using zeta potential, as shown in Supplementary Figure S3 and Supplementary Table S3. The size of DNA-bound PEI@IONPs (DNA/PEI@IONPs) was measured for 7 continuous days to test the nanoparticles’ stability. As DNA is negatively charged, after binding with PEI@IONPs, the positive surface charge on the PEI@IONPs drastically reduced, assuring successful binding of the plasmid DNA on PEI@IONPs via electrostatic interactions.

The TEM images of the IONPs of different shapes before and after PEI functionalization and DNA binding were found to retain the same morphologies (Figure S2). The zeta potential of the following nanoparticles showed positive values, indicating the presence of a PEI layer on the surface of the nanoparticles. The zeta potentials of the nanoparticles indicate that the nanoparticles disperse well in DD water since the electrostatic repulsive force among the nanoparticles is large, which gives the particles high aqueous stability under the conditions [37]. This positively charged PEI coating is important in binding negatively charged DNA for further gene delivery applications (Figure S4). The successful surface coating of PEI on the magnetic Fe_3_O_4_ nanoparticles is also confirmed via FTIR spectroscopy. The spectra of Fe_3_O_4_ before and after PEI coating display peak changes in different functional groups. PEI-modified Fe_3_O_4_ clearly shows a peak at ~3400 cm^−1^, which is attributed to the N-H stretching mode of the primary amine (Figure S5a) [38]. The wavenumber range of 585 cm^−1^ assigned to the Fe-O vibrations and these low band frequencies can be attributed to the spinel ferrite phase of Fe_3_O_4_, while the Fe-O band for γ-Fe_2_O_3_ is usually seen at 540 cm^−1^ [38]. Comparing the spectra before and after PEI coating, a sharp N-H bending peak can be seen for PEI-coated IONPs at a wavenumber of 1650 cm^−1^, also implying the presence of amine groups over IONPs. Similarly, there is a strong N-H signal peak for PEI@IONPs, i.e., nanorods (PEI@IONRs), nanospheres (PEI@IONSs), and truncated octahedrons (PEI@ITOHs), as well (Figure S5b).

The X-ray diffraction (XRD) pattern confirms the transformation of β-FeOOH to Fe_3_O_4_. The calculated lattice parameter of Fe_3_O_4_ nanorods (IONRs) is 8.39 Å, which agrees with the reported value (ICDD 19-629) (Figure S6a) [27]. The X-ray diffraction patterns match the literature values of magnetite crystals (JCPDS #: 070322) [39]; likewise, eight distinct peaks at 18.2, 30.1, 35.6, 37, 44.2, 53.6, 57, and 62.5° account for the crystal planes 111, 200, 311, 222, 400, 422, 511, and 440, respectively, which corresponds to the formation of magnetite (Fe_3_O_4_) nanocrystals in the given data [8]. For Fe_3_O_4_ truncated octahedrons (ITOHs), the powder XRD pattern of the structure also matches that of inverse spinel Fe_3_O_4_ with a face-centered cubic (fcc) structure, both in the position and in the relative intensity of all diffraction peaks (JCPDS NO.89-4319) (Figure S6b) [35]. In the XRD patterns for Fe_3_O_4_ nanospheres (IONSs), the sites and intensity of the diffraction peaks are consistent with the standard pattern for JCPDS (Card No. 79—0417) [40]. The sample shows very broad peaks at 220, 311, 400, 422, 511, and 440, indicating the ultra-fine nature and small crystallite size of the particles (Figure S6c) [40].

The room temperature hysteresis curves of IONRs, IONSs, and ITOHs were recorded to study the magnetic behaviors of IONPs (Figure S7). The saturation magnetization (M_s_) of IONRs (red) is 44.79 emu g^−1^ (electromagnetic unit per gram), which is smaller compared to the bulk value of Fe_3_O_4_ (92 emu g^−1^ for magnetite) [27]. In comparison to IONRs, the M_s_ value for IONSs (blue) is 60.25 emu g^−1^ and for ITOHs (green) is 109.35 emu g^−1^, which are higher. The lower M_s_ value for IONRs might be due to the existence of a surface-spi-disorder layer, which decreases with increasing particle diameter [27]; moreover, the shape anisotropy of the IONRs keeps the magnetizing characteristics in the same direction on their easy magnetic axis. The coercivity and remanence values are not visible at 300 K, indicating superparamagnetic behavior of the IONPs [27].

### 3.2. Preparation and Characterization of DNA/PEI@IONP Magnetoplexes for pDNA Delivery

The positively charged PEI@IONPs bind with the negatively charged pDNA, forming magnetoplexes of DNA/PEI@IONPs, which were determined using the DNA retardation test via agarose gel electrophoresis (Figure S4). The magnetoplexes were formed using different weight ratios (*W/W*) of PEI@IONPs (15, 10, 5, 3, 1, 0.5) combined with a fixed pDNA amount (3 µg). The results show that at a weight ratio of >3 onwards, the nanoparticles can encapsulate DNA via electrostatic interactions and can be retained inside the well. To deliver a gene inside cells, the compaction of pDNA with PEI@IONPs plays an important role in the success of the endocytosis process, and the particle size required is up to 150 nm in diameter [41]. To investigate the level of compaction of pDNA binding with PEI@IONPs, the diameters of the DNA/PEI@IONPs were measured and compared with the size of the PEI@IONPs without pDNA binding at three different W/W ratios (5, 10, 15) (Table S2). Owing to the presence of PEI on the surface of IONPs, a stronger electrostatic interaction developed between the PEI@IONPs and pDNA, resulting in a decrease in the sizes of the magnetoplexes compared to those without pDNA on the surface of PEI@IONPs [41]. It was also found that when magnetoplexes were made as per the weight ratio, the size of the magnetoplexes increased gradually with increasing weight ratios of 5, 10, and 15, confirming the increasing binding amount of PEI@IONPs with fixed pDNA. The average mean particle size of DNA/PEI@IONPs in all the weight ratios was in the range of 50 to 150 nm, which favors good transfection efficiency of the nanoparticles across the cell membranes [41].

### 3.3. Cytotoxicity of PEI@IONPs and DNA/PEI@IONPs

**Figure 2:**
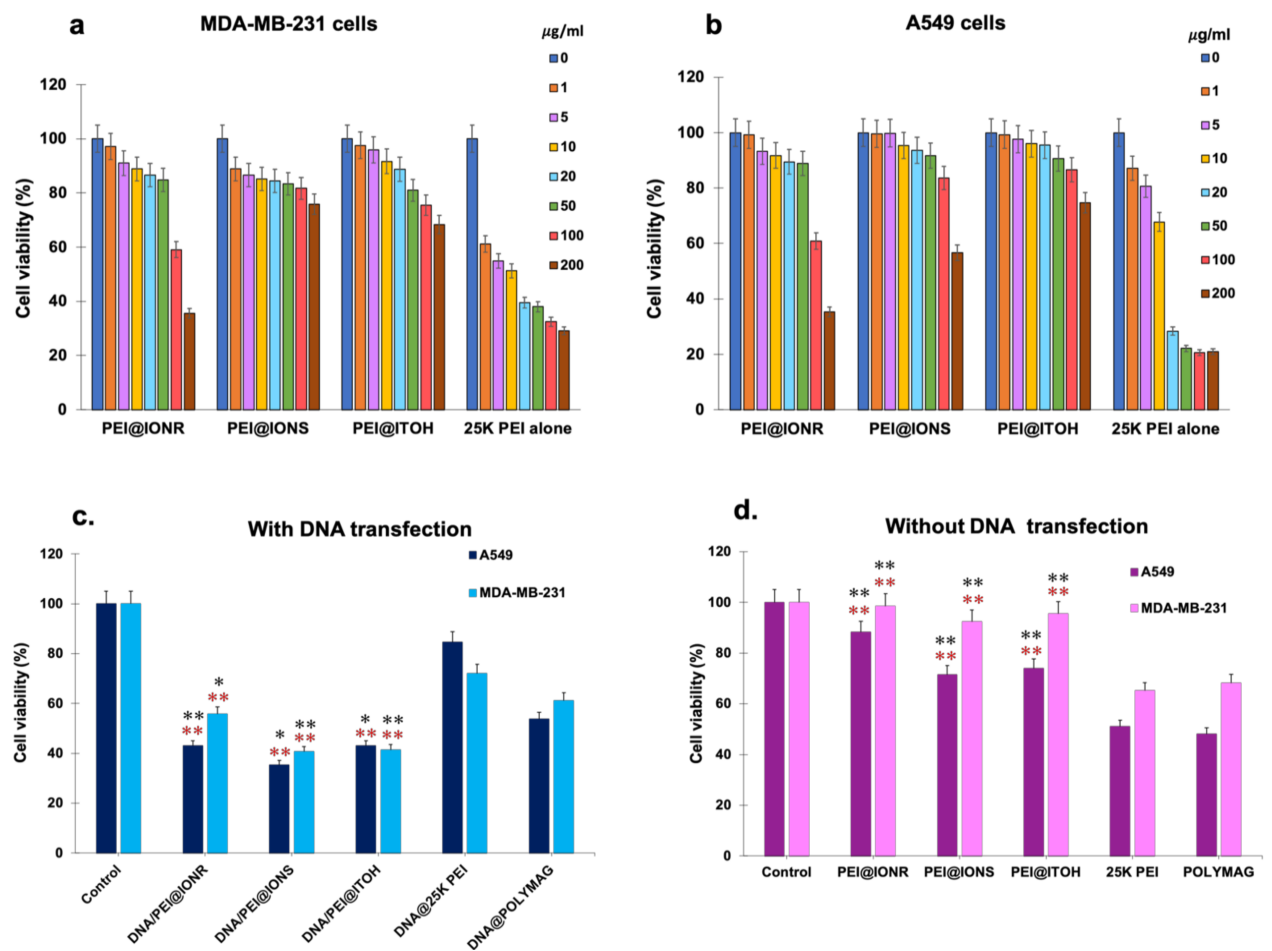
**Percentage of cell viability with and without miRNA34a transfection.** Cell viability of (**a**) MDA-MB-231 and (**b**) A549 after 48 hours of incubation with PEI-functionalized iron oxide nanoparticles (PEI@IONPs) of different morphologies at different concentrations ranging from 0 to 200 𝜇g/mL. The percentage of cell viability was compared with the cells alone, i.e., 0 𝜇g/mL. The data are expressed as mean ± standard deviation (*n* = 8). (**c**) The percentage of cell viability after transfection with miRNA34a and without miRNA34a for 48 hours with PEI@IONPs of different morphologies at a weight ratio of 5 (*w/w* = 5). After miRNA34a transfection, the percentage of cell viability decreased drastically in the cells treated with PEI@IONPs compared to PEI 25K (_black_) and PolyMag (_red_). (**d**) Without miRNA34a transfection, the cell viability was higher with PEI@IONPs compared to PEI 25K _(black_) and PolyMag (_red_). The data are expressed as mean ± standard deviation (*n* = 3, * *p* < 0.05, ** *p* < 0.01)

We checked the cytotoxicity of PEI@IONPs in comparison to 25,000-branched PEI (25K B-PEI) in A549 and MDA-MB-231 cells at different concentrations (Figure 2a,b). No obvious cytotoxicity of PEI@IONPs was observed for increasing concentrations ranging from 0 to 200 μg/mL in cell lines; on the other hand, the gold-standard transfection reagent PEI 25K alone was found to have cytotoxicity in cells with increasing concentration. Generally, the higher-molecular-weight PEI derivatives were shown to have increased toxicity compared to low-molecular-weight PEI [40]. PEI on its own exhibits some cytotoxicity [42], and the negligible or lower cytotoxicity of PEI@IONPs suggests that the polymer stays intact on the surface of the nanoparticles in cell media, which reduces the cationic and toxic charge when the PEI is adsorbed on the surface [43].

We further evaluated the cytotoxicity of PEI@IONPs after transfecting them with and without miRNA34a-induced pDNA at a weight ratio of 5 after 48 h of incubation. The percentage of cell viability is lower in cells treated with DNA/PEI@IONPs compared to PEI 25K and PolyMag (Figure 2c). PEI alone shows cytotoxicity, but interestingly, after binding with DNA, it does not show much cytotoxicity [43]. PolyMag, which is also cytotoxic, shows a further decrease in cell viability after binding with DNA. The cells transfected without plasmid DNA show higher percentages of cell viability with PEI@IONPs compared to PEI 25K and PolyMag (Figure 2d). As mentioned above, PEI 25K alone is cytotoxic at higher concentrations, but upon complexation with nanoparticles as observed, its cytotoxicity drops [42]. The cells incubated with PolyMag were detached and clumped, which is a clear indication of induced damage, and this cytotoxicity is most likely associated with the presence of polycations [44]. These results indicate that PEI@IONPs alone cause no significant harm to the cells and can be considered safe delivery agents inside the tumor microenvironment.

According to studies, miRNA34a overexpression in cancer cells reduces cellular viability by inducing apoptosis [20]. In this study, in the case of PEI@IONPs, the apoptosis rate is higher due to higher transfection efficiency, leading to lower cell viability compared to PEI 25K and PolyMag. As PolyMag alone shows higher cytotoxicity, the cells tend to die due to cytotoxicity along with apoptosis, and thus, lower cell viability is seen. PEI is cytotoxic, but after binding with DNA, the cytotoxicity is reduced and transfection efficiency with PEI 25K is found to be reduced when compared to PEI@IONPs.

### 3.4. In Vitro Gene Transfection of miRNA34a Using PEI@IONPS

**Figure 3:**
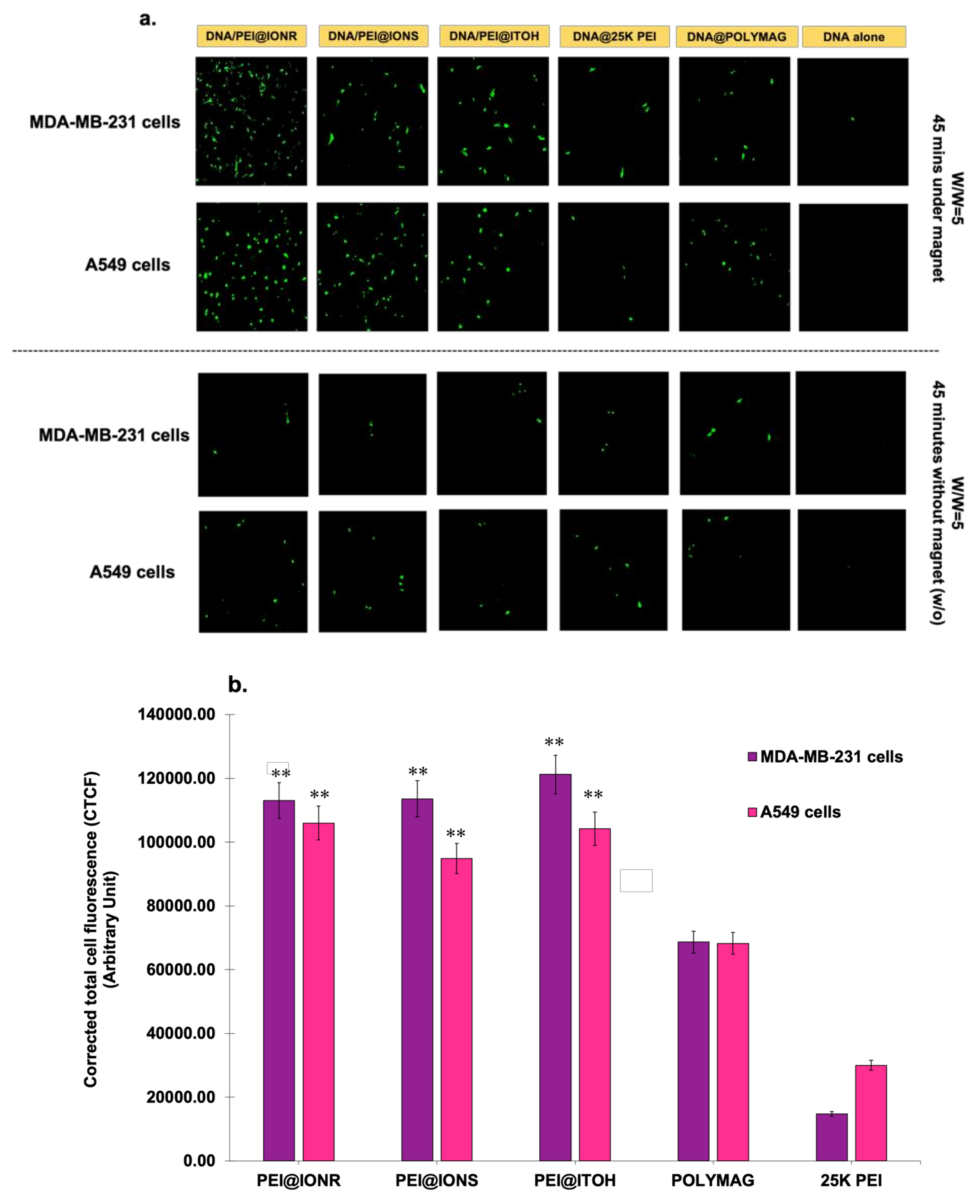
**Transfection efficiency study, involving the delivery of plasmid DNA, using PEI@IONPs.** (**a**) Expression of green fluorescent protein (GFP), observed through fluorescence microscope in MDA-MB-231 and A549 cell lines transfected with GFP-tagged miRNA34a intact pDNA using PEI@IONPs and incubated with and without (w/o) a static magnet, for 45 min, at a DNA/IONP weight ratio of 5 (*W/W* = 5), compared with PolyMag as a positive control. (**b**) Corrected total cell fluorescence (CTCF) of MDA-MB-231 and A549 cells calculated using ImageJ software. The results are represented as mean value ± standard deviation (*n* = 3, ** *p* < 0.01).

With the aim of establishing an effective therapeutic protocol, we confirmed successful entry of the nanoparticles inside the cells. To investigate this, the cells were exposed to PEI@IONPs encapsulated by pDNA (DNA/PEI@IONPs) encoding miRNA34a and the GFP reporter at a weight ratio 5, and subjected to a static magnetic field for 45 min in an incubator. A total of 48-h post-incubation, the intensities of GFP expression in the cells transfected with DNA/PEI@IONPs were found to be significantly higher than in those transfected with the gold standard reagent 25K PEI and the commercially available Magnetofection^TM^ reagent PolyMag (Figure 3a). Statistical analysis was conducted using ImageJ, and the CTCF (corrected total cell fluorescence) values were calculated for each treated group. Figure 3b shows that cells readily uptake DNA/PEI@IONPs as compared to PolyMag under a static magnet. PEI 25K alone is taken as a negative control as it does not carry a magnetic property, so the transfection efficiency is lowest compared to the PEI@IONPs and PolyMag. Nevertheless, the higher cytotoxicity of PolyMag (Figure 2d) also results in poorer transfection; thus, we tentatively confirm that the magneto-gene carrier is more efficient and its gene delivery capacity is further enhanced due to the magnetic nature that assists with deeper penetration inside the cells. The further internalization of nanoparticles in the cytosol takes place under the proton sponge effect due to the presence of PEI on the nanoparticles’ surface [36].

**Figure 4:**
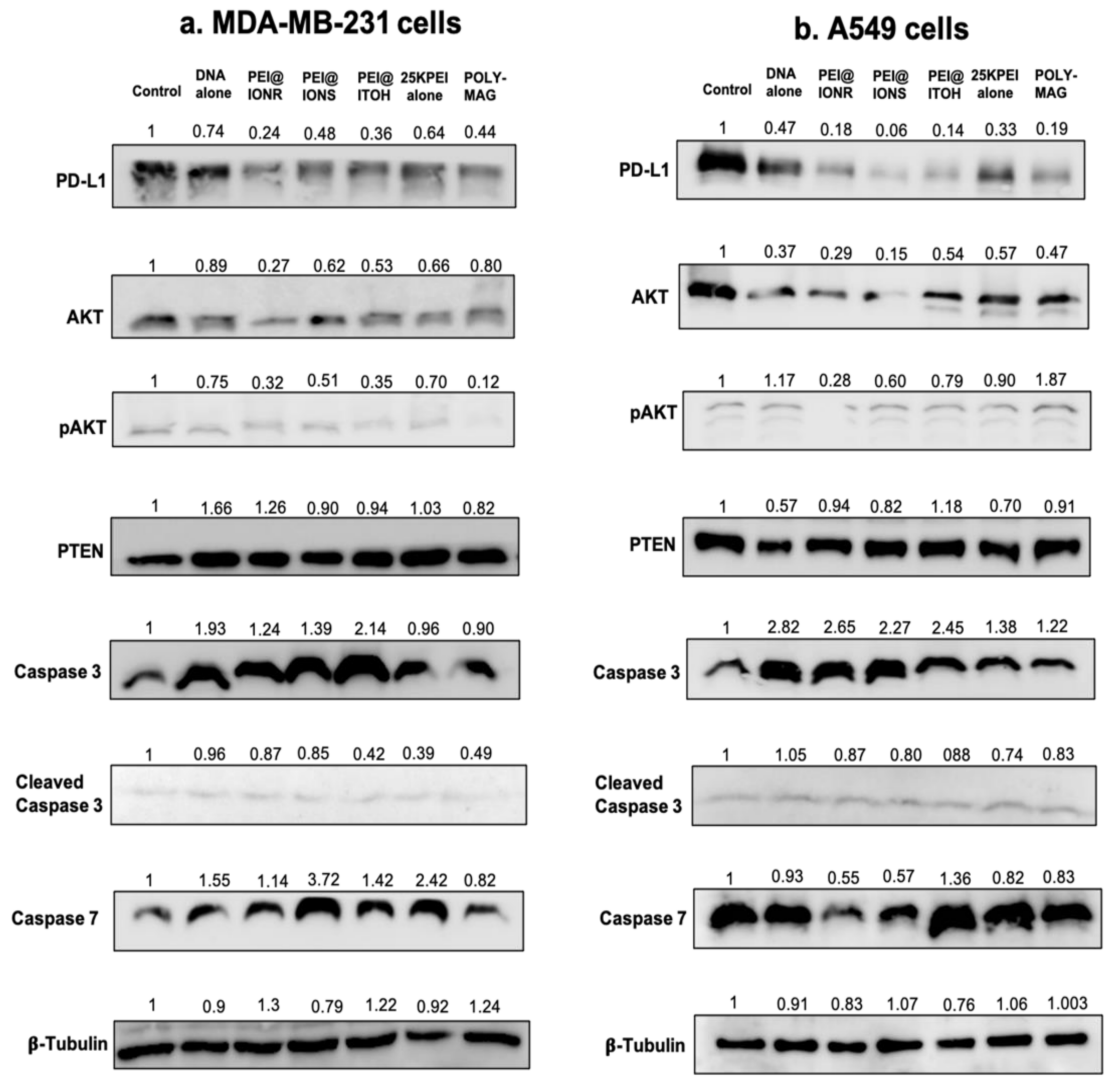
**Western blot analysis for PD-L1 protein and its signaling pathways**. After transfection with miRNA34a in the (**a**) MDA-MB-231 and (**b**) A549 cell lines, the results show the success and higher percentages of knockdown in PD-L1 protein levels after treatment with PEI@IONPs compared to PEI 25K alone. PD-L1 silencing is also seen with PolyMag treatment, mostly due to the cell cytotoxicity after treatment leading to lower concentrations of protein lysate. The signaling proteins AKT and pAKT are downregulated as they are responsible for promoting and regulating PD-L1 in cancer cells. PTEN is seen to be upregulated as it acts as a tumor suppressor for the PD-L1 gene. The apoptosis markers caspase 3, cleaved caspase 3, and caspase 7 show upregulation after transfection with miRNA34a/PEI@IONPs, inducing apoptosis in cancer cells. Protein expression was analyzed via ImageJ using the fold change method.

For the functional evaluation of PD-L1 in A549 and MDA-MB-231 cells transfected with miRNA34a/PEI@IONPs, immunoblot analysis was performed using antibodies to detect the PD-L1 protein and signaling pathways. The Western blot data in Figure 4 show significant reduction in the levels of PD-L1 in the cells treated with PEI@IONP-encapsulated miRNA34a. Nevertheless, the cells treated with PEI 25K do not show much reduction in PD-L1 protein expression; however, after treatment with PolyMag, cell loss is observed due to cytotoxicity, resulting in lower lysate formation and a lower protein concentration. A reduction in PD-L1 protein levels is also seen with PolyMag treatment, affirming the functional knockdown of the PD-L1 gene by miRNA34a. We also investigated the signaling pathways in PD-L1 gene regulation and promotion. The roles of proliferation factors such as AKT and phosphorylated AKT (pAKT) were assayed [45]. AKT and pAKT are oncogenic proteins responsible for the regulation and deregulation of cell survival, growth, proliferation, and apoptosis [46]. The AKT marker is seen to be overactivated in malignancies, while pAKT is seen to be elevated in patients, particularly those with TNBC and NSCLC [46]. As can be seen in Figure 4, AKT and pAKT show significant downregulation in the cells treated with PEI@IONP-encapsulated miRNA34a; however, when compared to PolyMag, the knockdown level is also reduced due to the loss of cells, as mentioned above. The tumor suppressor gene PTEN was also evaluated as loss of PTEN expression can promote tumor progression, and it also acts as a mechanism responsible for resistance to anti PD-1/PD-L1 treatment [47]. Accordingly, PTEN shows significant upregulation in the cells after transfection with miRNA34a using PEI@IONPs, indicating tumor suppression in the cell lines. Though the cytotoxic behavior of PolyMag affects the protein levels’ ability to produce a satisfactory outcome, this study validates that PEI@IONPs have the potential to deliver miRNA34a for significant silencing of the PD-L1 genes.

### 3.5. Apoptosis after Transfection with miRNA34a/PEI@IONPs

**Figure 5:**
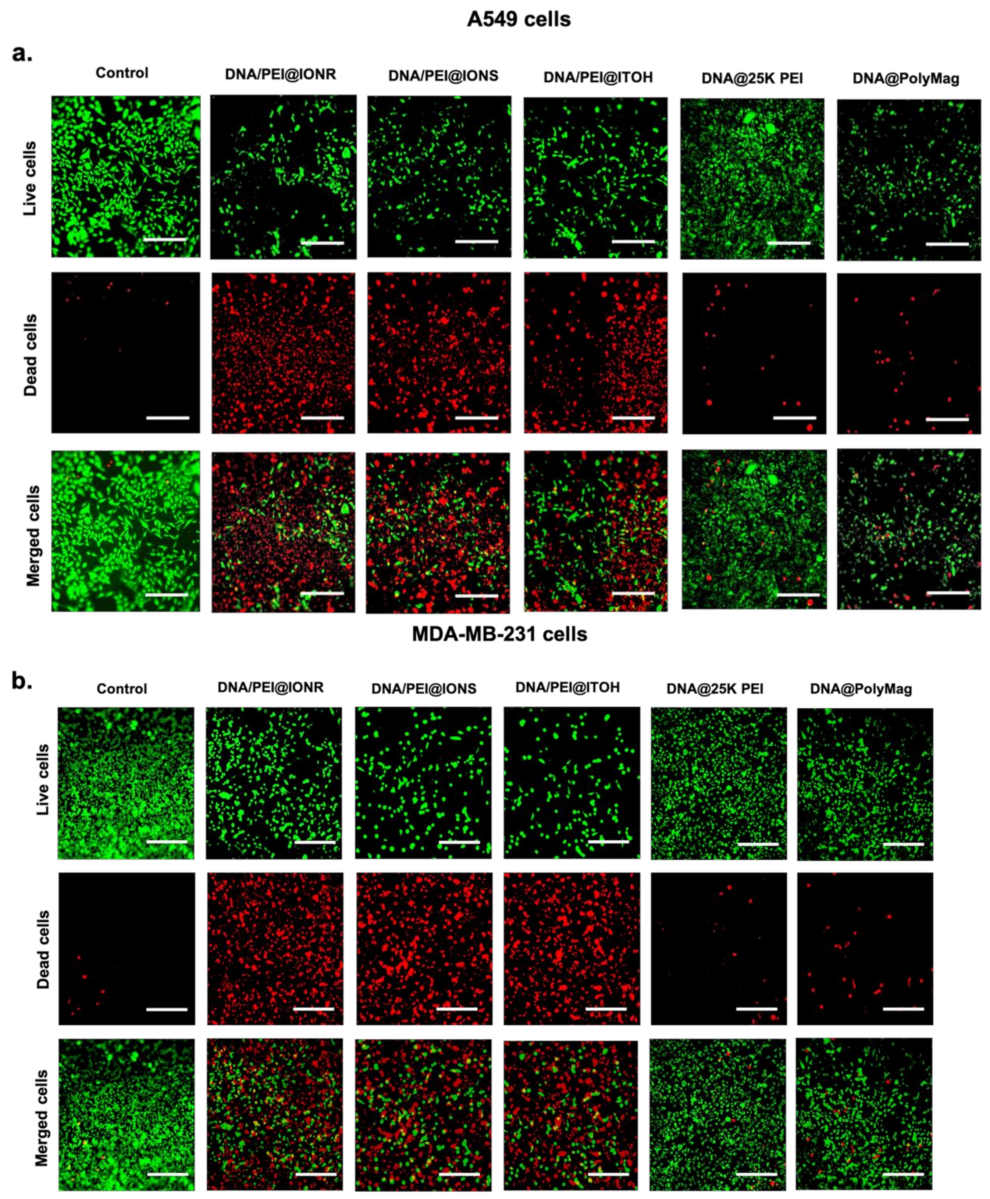
Live/dead cell staining. (**a**) A549 and (**b**) MDA-MB-231 cells transfected with miRNA34a and incubated for 48 hours. Post-transfection cells were stained with calcein-AM/PI staining solution and images were captured using a fluorescence microscope. Scale bar: 100 μm. Cells stained in green indicate live cells and cells stained in red indicate dead cells, probably undergoing apoptosis after transfection. In contrast, cells treated with PEI 25K and PolyMag showed higher cytotoxicity, which caused surface detachment during the staining process after washing with phosphate-buffered saline (PBS), leading to cell loss.

Cells were visualized through live/dead staining with calcein-AM/propidium iodide (PI) after transfection with miRNA34a using DNA/PEI@IONPs magnetoplexes. It is well-known that cell death occurs via two mechanisms: either apoptosis or necrosis [48]. After treatment with calcein-AM/PI, the live cells were stained with green, and dead cells with red, respectively. In Figure 5, the cells treated with PEI@IONPs show more red-stained cells compared to green-stained cells. On the other hand, the cells treated with commercial the transfection reagents PEI 25K and PolyMag show more green cells compared to red cells. Some of the cells are also seen in a yellow color, and the presence of a bright green or yellowish stain denotes that cells are undergoing early apoptosis, whereas cells with a red stain indicate late apoptosis [48]. This result confirms that after transfection with miRNA34a, cells undergo apoptosis, which is revealed more efficiently using DNA/PEI@IONPs magnetoplexes than using the commercially available transfection reagents. Both PEI 25K and PolyMag demonstrate high cytotoxicity, as shown in the biocompatibility assay (Figure 2c,d), but contradictorily, we observe less cytotoxicity under treatment with PEI 25K. This may be attributed to the short exposure of PEI for only 45 min, followed by washing of the cells, which removed the minor traces of toxic PEI from the culture media, leading to fewer apoptotic cells being harvested [44].

**Figure 6:**
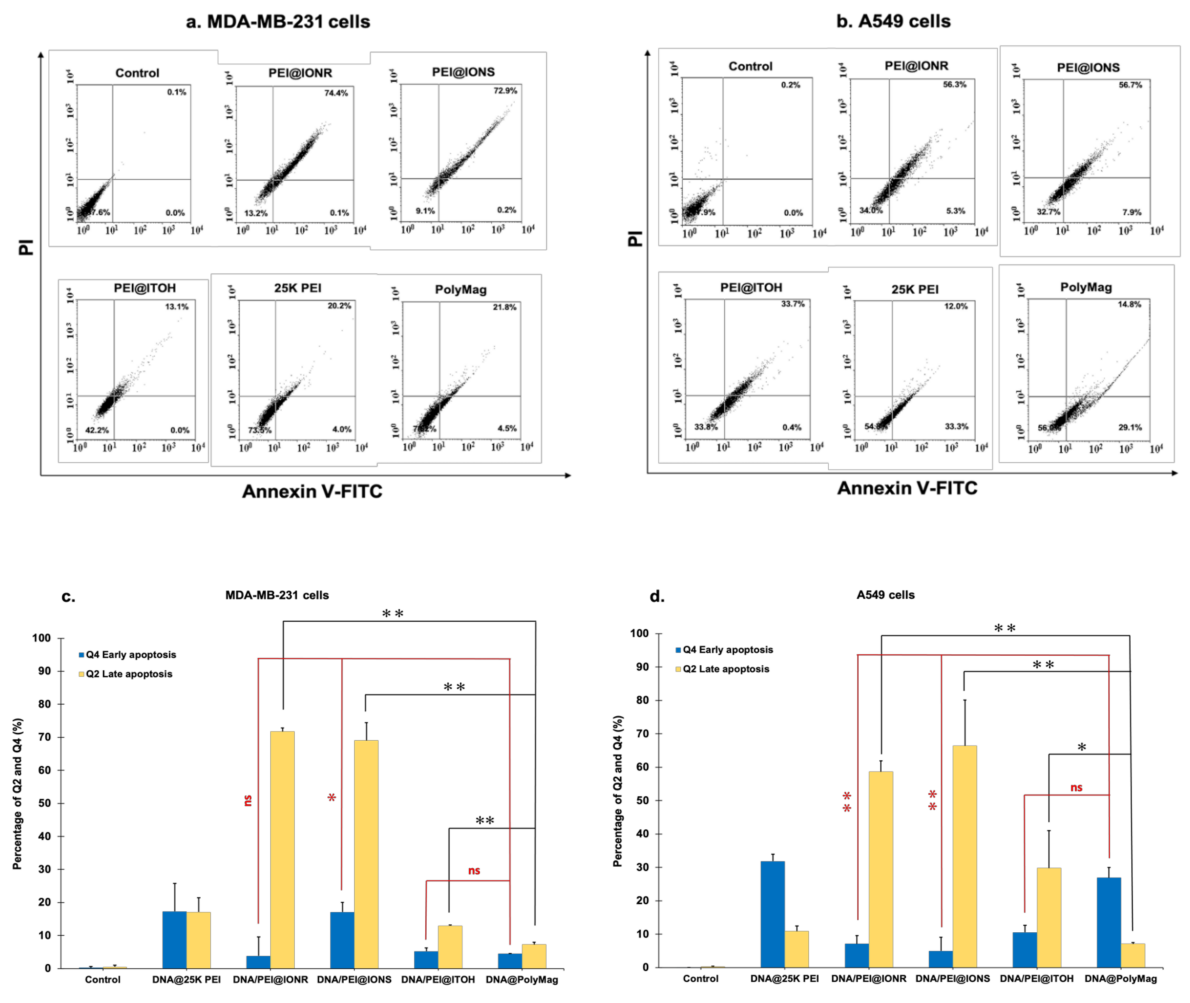
**Flow cytometry analysis to study cell apoptosis.** After transfection with miRNA34a/PEI@IONPs at a weight ratio of 5 in (**a**) MDA-MB-231 and (**b**) A549 cells, flow cytometry analysis showed that the maximum population of cells was located in late apoptosis quadrant Q2 (the upper right corner), indicating the capability of miRNA34a to induce apoptosis in cancer cells. The quantification of flow cytometry percentages in early apoptosis and late apoptosis was performed after transfection of miRNA34a for 48 hours in (**c**) MDA-MB-231 and (**d**) A549 cell lines and compared to PolyMag as a positive control. The results are represented as mean value ± standard deviation (*n* = 3, * *p* < 0.05, ** *p* < 0.01).

In the apoptotic process, the initiator caspase is activated, which cleaves and activates downstream executioner caspases that further cleave subsequent proteins. Therefore, the activation of executioner caspases is considered to be a sign of apoptotic cells [48]. With reference to live/dead cell studies, to further validate that the cells are undergoing an apoptosis process, flow cytometry was performed using a dual staining approach with annexin V-FITC and PI. The cells transfected with miRNA34a/PEI@IONPs reveal higher percentages of apoptosis compared to PolyMag- and PEI 25K-treated cells (Figure 6). This result coincides with the fluorescent cell images showing dead cells in the live/dead studies (Figure 5) and the low cell viability found in the MTT assay (Figure 2c). Western blot analysis of the apoptotic proteins shows the upregulation of caspase 3, cleaved caspase 3, and caspase 7, which further supports the cells having died due to apoptosis (Figure 4). We conclude that PEI@IONPs have high transfection efficiency for the delivery of miRNA34a-encoded plasmid DNA, which induces the apoptosis process along with PD-L1 gene knockdown.

### 3.6. miRNA34a Expression Using Real-Time Quantitative Polymerase Chain Reaction (qRT-PCR)

The successful transfection of miRNA34a into cells using PEI@IONPs, as visualized using fluorescence microscopy and knockdown of the PD-L1 gene, was further confirmed via Western blotting; then, we investigated the miRNA34a expression levels inside cells using real-time quantitative polymerase chain reaction (qRT-PCR) after transfecting A549 and MDA-MB-231 cells, and compared the efficiency of nanoparticles with other commercially available transfection reagents. We selected PEI@IONRs for the qRT-PCR study as they showed highest transfection efficiency compared to PEI@IONSs and PEI@ITOHs. The qRT-PCR result shows that the miRNA34a expression levels were higher in the cells transfected with miRNA34a/PEI@IONRs compared to the commercial transfection reagent PolyMag (Figure S8). This is possibly due to the higher surface area of nanorods, enabling them to bind more plasmid DNA, and their anisotropic morphology, which induces a stronger magnetic field to allow for better transfection in the cells [27].

## 4. Conclusions

The summary of our work shows that we successfully synthesized iron oxide nanoparticles of different shapes and sizes and fabricated their surfaces with branched PEI 10K to give them a stable shape and positive surface charge. The positively charged PEI-coated nanoparticles were capable of binding negatively charged plasmid DNA via electrostatic interactions, which gave further ease to the delivery of plasmid DNA inside cells. The cytotoxicity of IONPs was less than with PEI and PolyMag. We successfully transfected pDNA-encoded miRNA34a into the A549 and MDA-MB-231 cells, with the assistance of IONPs using a static magnet, which targeted the PD-L1 gene and led to its silencing. The transfection of miRNA34a and the silencing of PD-L1 genes were confirmed via fluorescence microscopy and Western blotting studies. The therapeutic study using live/dead cells was also successful with IONPs, and showed higher apoptosis. The gene expression study of miRNA34a also showed higher expression in the cells treated with nanorods compared to PolyMag. Among all three IONPs, IONRs were the most efficient transfection agents compared to IONSs and ITOHs. In conclusion, the unique concept of delivering miRNA34a using PEI@IONPs proves that IONPs hold the potential to deliver different genes under magnetofection, as IONPs are magnetic in nature, thereby giving them the advantage to enter target cancer cells. IONPs have already demonstrated a wide impact on the health sector with their potential to gain deep access to cell systems while possessing negligible cytotoxicity. With their ease of synthesis and flexibility in their shapes and sizes, IONPs can be directed toward the target disease sites due to their magnetic properties, and with our unique strategy of delivering genes, there is wide scope to investigate more target genes and discover possible treatments for different types and a wider range of cancer diseases in the future.

## Data Availability

Not applicable.

## Supplementary Materials

The following are available online at https://www.mdpi.com/article/10.3390/pharmaceutics15010215/s1, Figure S1: Reaction scheme of IONR; Figure S2: TEM images of IONPs with different morphologies; Figure S3: Size stability test of DNA/PEI@IONPs; Figure S4: Agarose gel electrophoresis of PEI@IONPs; Figure S5: FTIR images of Fe3O4 NPs and PEI@IONPs; Figure S6: XRD patterns of IONPs; Figure S7: Magnetization curves for different shapes PEI@IONPs; Figure S8: miRNA34a expression levels in MDA-MB-231 and A549 cells; Table S1: Hydrodynamic sizes and zeta potentials of IONPs; Table S2: Hydrodynamic size of PEI@IONPs and DNA/PEI@IONPs; Table S3: Zeta potentials DNA/PEI@IONPs.
