# Supplementary material for: Comparing the Variants of Iron Oxide Nanoparticle-Mediated Delivery of miRNA34a for Efficiency in Silencing of PD-L1 Genes in Cancer Cells
Source: Pharmaceutics. 2023 Jan 8;15(1):215. doi: 10.3390/pharmaceutics15010215 (PMC9865708; doi:10.3390/pharmaceutics15010215)

pharmaceutics

MDPI

Supplementary Materials: Comparing the variants of iron oxide nanoparticles mediated efficient delivery of miRNA34a for effective silencing of PD-L1 genes in cancer cells

Richa Pandey, Feng-Shuo Yang, Vyshnav Sivasankaran, Yu-Lun Lo , Yi-Ting Wu, Chia-Yu Chang, Chien-Chih Chiu, Zi-Xian Liao and Li-Fang Wang

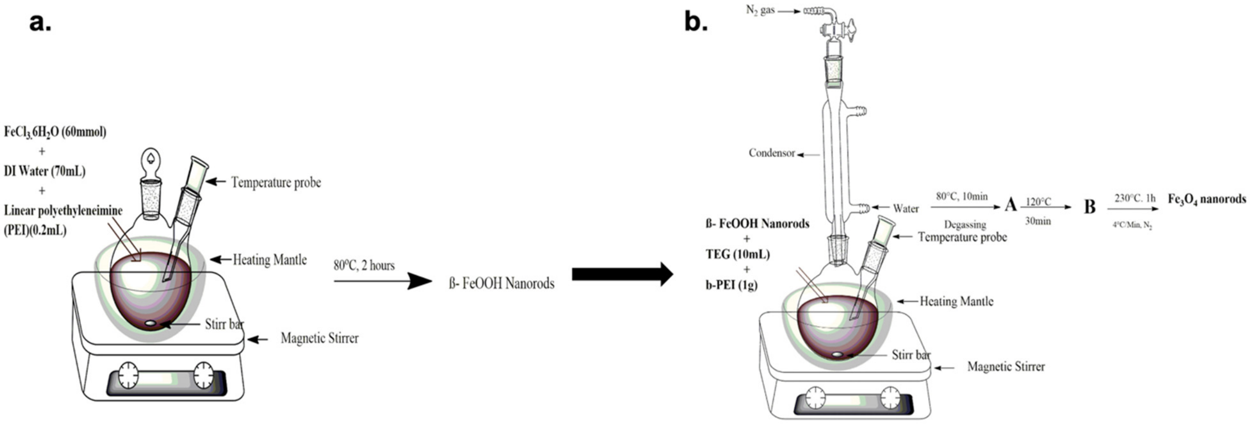

Supplementary Figure S1 | Reaction scheme of the two step synthesis reaction for iron oxide (Fe_3O_4) nanorods (IONR). (**a**) Hydrolysis of Iron(III) chloride ($FeCl_3$) into β-FeOOH nanorods structure of uniform sizes formed with a mean length of 70 nm. (**b**) β-FeOOH nanorods were reduced to the iron oxide (Fe_3O_4) using tetraethylene glycol (TEG) as a reducing agent at 230°C where the rods shape and size are retained.

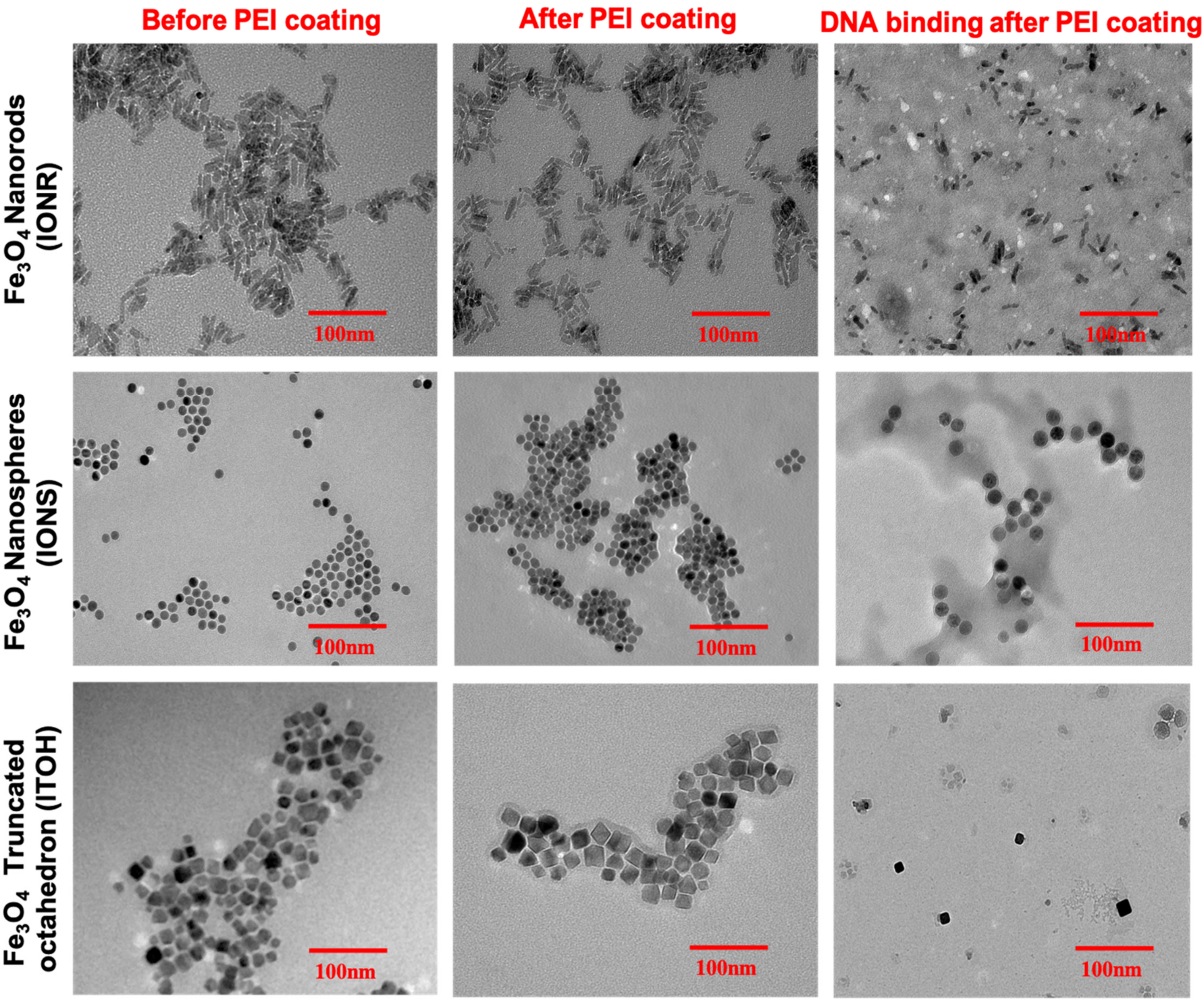

Supplementary Figure S2 | TEM images of the Fe_3O_4 nanoparticles (IONPs) of different morphologies after surface functionalization and DNA binding. Fe_3O_4 nanorods, nanospheres and truncated octahedrons after surface functionalization with branched PEI (B-PEI) show no significant difference in shape and size of the nanoparticles before PEI coating, indicating the stability of the nanoparticles. The PEI coated IONPs (PEI@IONPs) is further subjected to DNA binding as DNA is negatively charged and can bind on the positive surface of nanoparticles due to the presence of the PEI via electrostatic interactions.(Scale bar: 100nm).

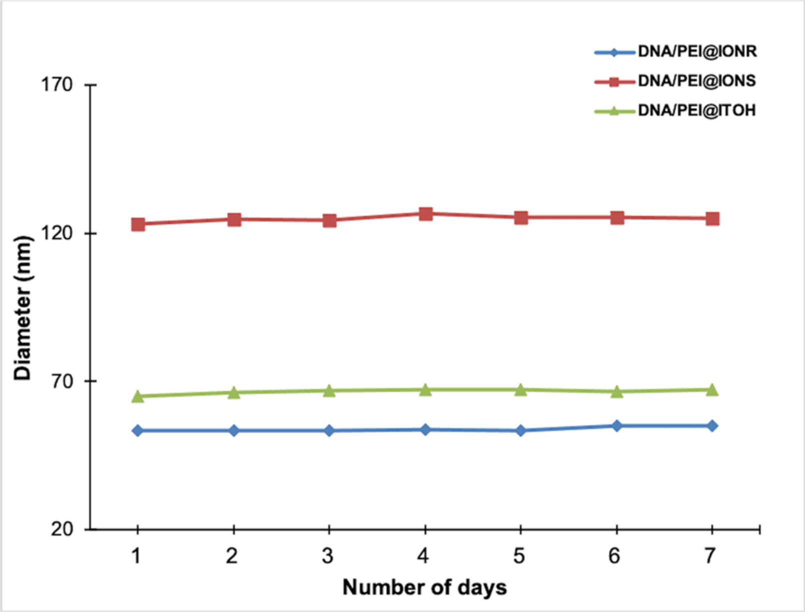

Supplementary Figure S3. | Size stability test of DNA/PEI@IONPs: The hydrodynamic sizes of DNA bound PEI@IONPs (DNA/PEI@IONPs) done for 7 continuous days. The result shows almost stable size of all the three nanoparticles carrying plasmid DNA.

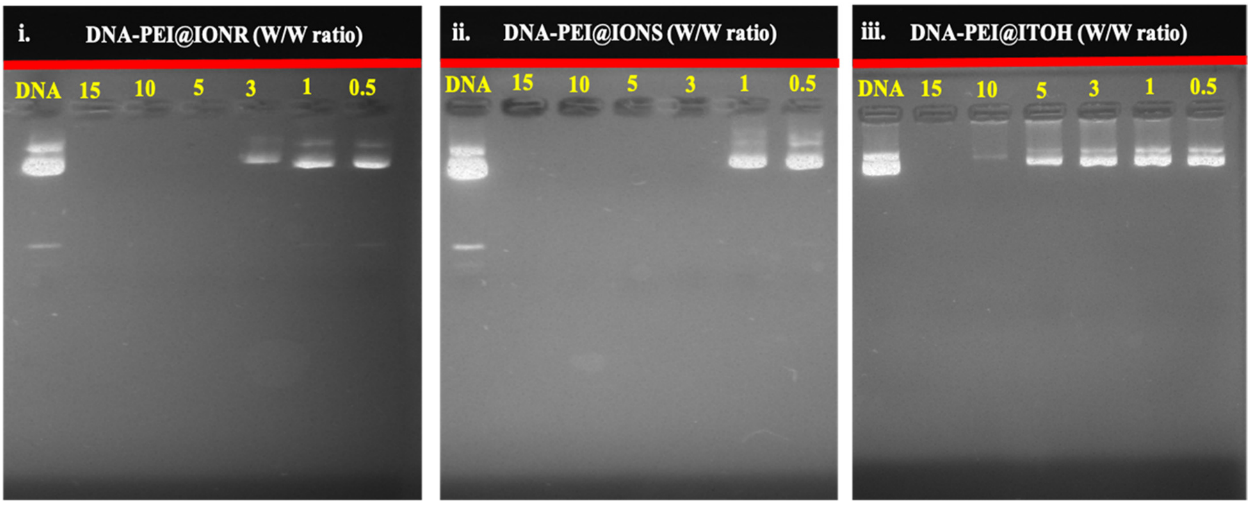

Supplementary Figure S4. | Preparation PEI coated IONPs (PEI@IONPs) and plasmid DNA magnetoplexes (DNA/PEI@IONPs). Agarose gel electrophoresis study for DNA retention test show the binding of pDNA with PEI@IONPs at different weight ratios. The most intact binding of pDNA is at the weight ratio of ≥3 due to electrostatic interactions between positively charged PEI on the surface of IONPs and negatively charged pDNA.

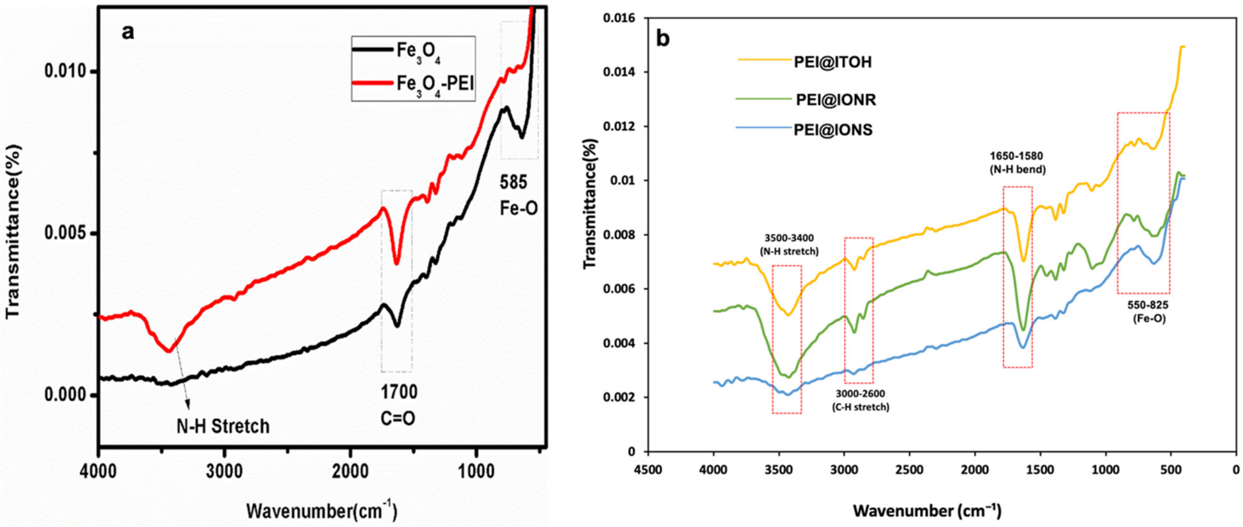

Supplementary Figure S5. | FTIR images of Fe_3O_4 nanoparticles. (a) Before and after coating with 10K branched PEI which show a sharp N-H stretch with PEI coated nanoparticles indicating the presence of amine group of the surface of Fe_3O_4 nanoparticles. (b) Surface functional groups for PEI@IONR (green), PEI@IONS (blue) and PEI@ITOH (yellow), which again show N-H stretch and bend indicating the successful PEI coating on the surface of the IONPs. From the comparative analysis the differences in the peaks obtained after coating with PEI can be seen clearly.

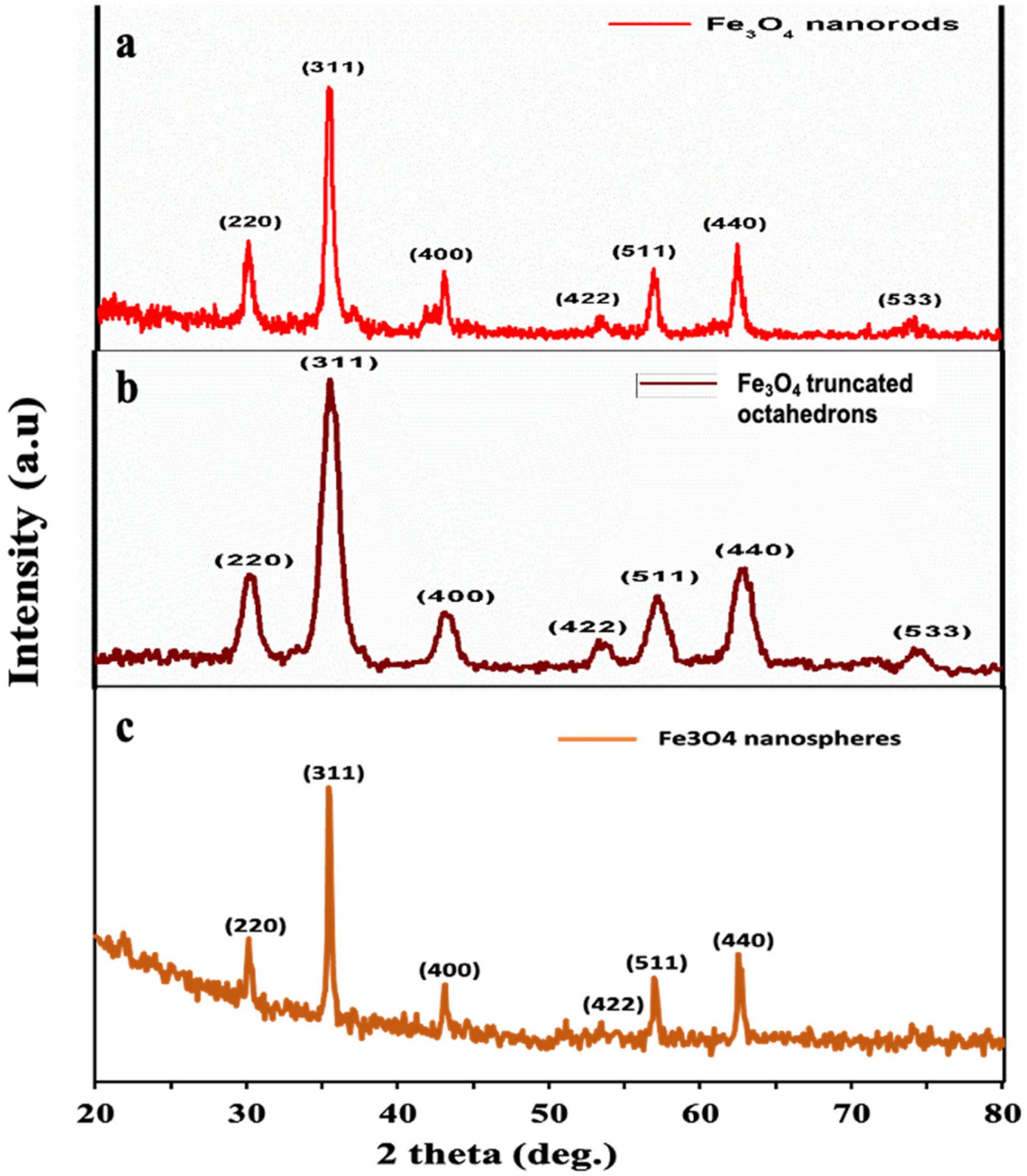

Supplementary Figure S6. | X-ray diffraction (XRD) patterns of (a)Fe_3O_4 nanorods, (b)Fe_3O_4 truncated octahedrons and (c)Fe_3O_4 nanospheres. The XRD patterns are labelled with reference to the standard ICDD cards.

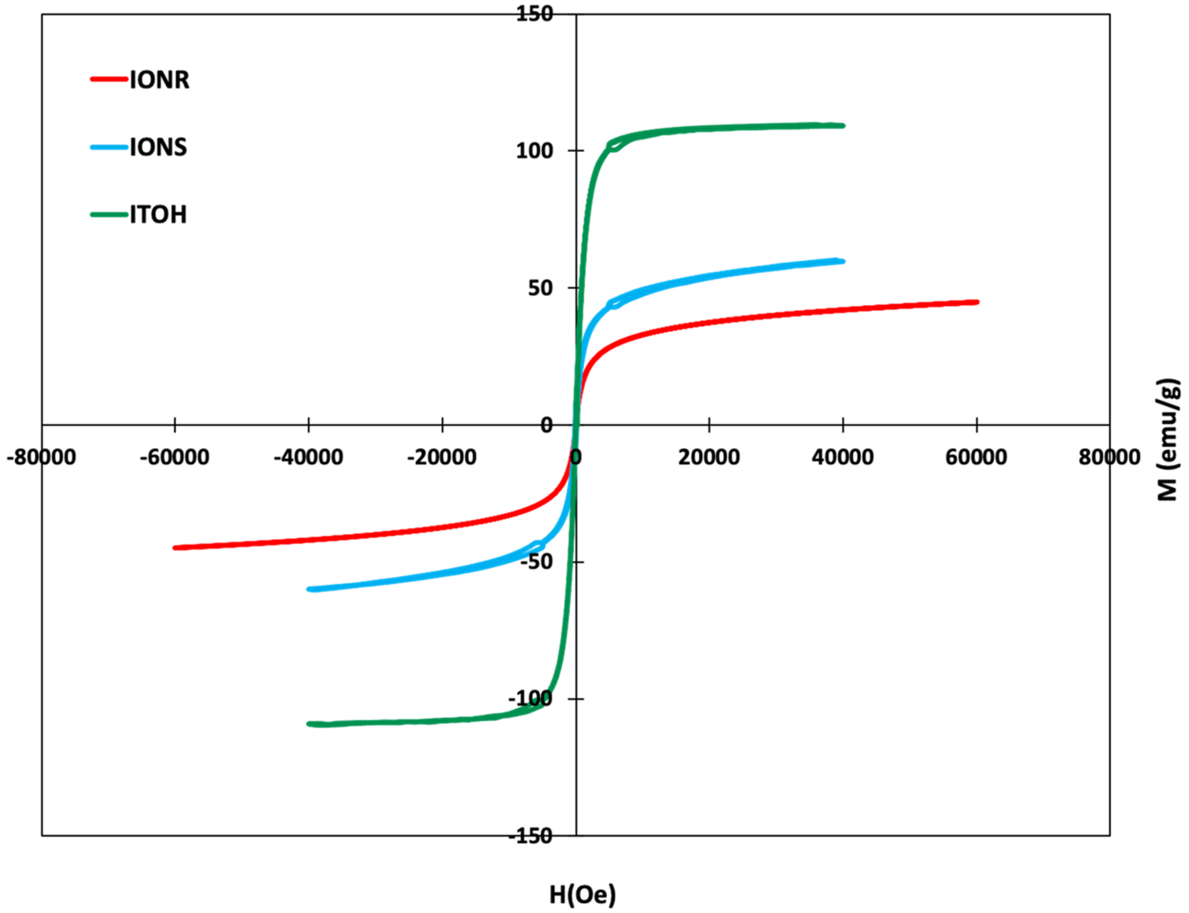

Supplementary Figure S7. | **Magnetization curve analysis for different shapes PEI@IONPs**. Magnetization curves obtained at 300K temperature of PEI coated Fe_3O_4 such as nanorods IONR (red), nanospheres IONS (blue) and truncated octahedrons ITOH (green). M represents mass magnetization (emu/g) versus H which represents applied magnetic field (Oe).

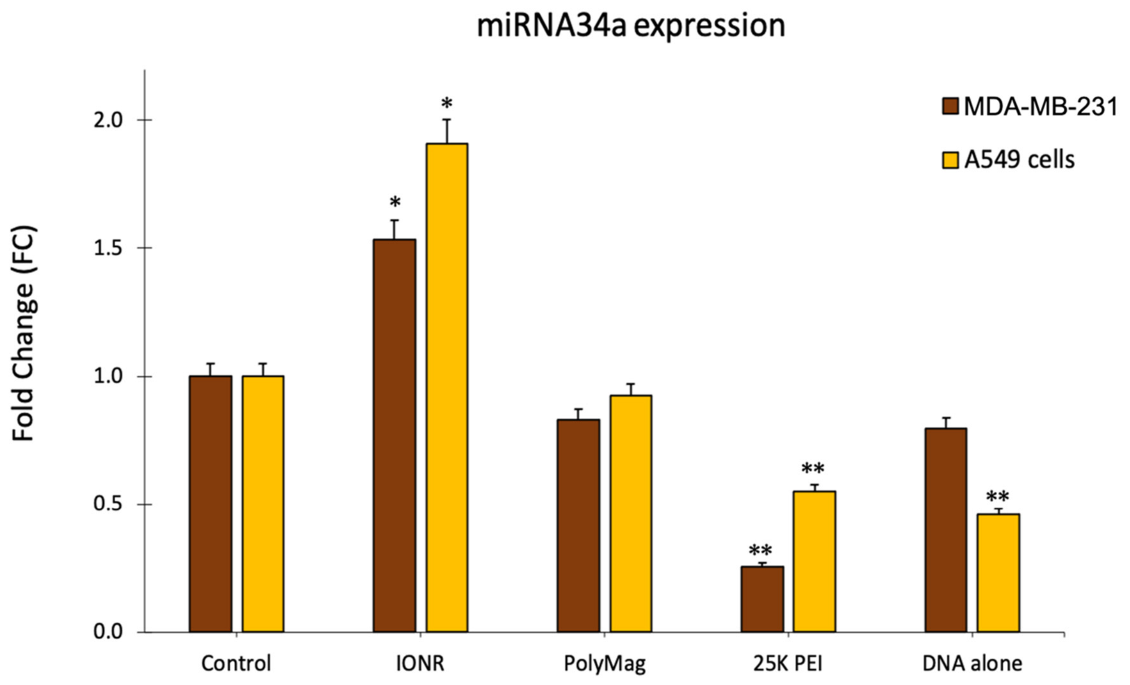

Supplementary Figure S8. | **miRNA34a expression levels** in MDA-MB-231 and A549 cell lines after transfection with PEI@IONR and positive control PEI and PolyMag, determined by qRT-PCR. PEI@IONR show higher expression of miRNA34a as compared to PolyMag. The data is represented as mean value ± SD (n=3, *p<0.05, **p<0.01).

Table S1. Hydrodynamic size (diameter size; PDI: polydispersity index) and zeta potentials of nanoparticles before and after PEI coating (means±SD, n=3). The iron (Fe) ion concentration was also measured using ppm (means±SD, n=3).

Samples	Size (nm) (Before PEI coating) Mean±SD	Polydispersity Index (PDI) Mean±SD	Size (nm) (After PEI coating) Mean±SD	Polydispersity Index (PDI) Mean±SD	Zeta Potential(mV) Mean±SD	Fe content (ppm) Mean±SD
IONR	95.57±0.153	0.22±0.014	154±0.96	0.17±0.009	54.69±0.27	1.135±0.007
IONS	167.4±11.84	0.40±0.008	185.5±2.35	0.18±0.006	48.98±0.53	1.167±0.0018
ITOH	126.3±3.48	0.22±0.005	132.9±2.85	0.19±0.02	44.86±0.75	1.116±0.0008

Table S2. Comparative hydrodynamic size (diameter size; PDI: polydispersity index) of PEI@IONPs and DNA/PEI@IONPs at different weight ratios 5, 10 and 15, respectively (n=3; mean±SD).

**Samples without DNA (W/O)**	**Size before DNA binding (nm)**	**Polydispersity Index (PDI)±SD**	Samples with DNA	Size (nm) after DNA binding at different weight ratios (W/W) (Mean±SD)					
				5	PDI±SD	10	PDI±SD	15	PDI±SD
**PEI@IONR**	**154±0.96**	**0.17± 0.009**	DNA/PEI@IONR	54.23±1.07	0.314±0.016	55.63±0.87	0.345±0.004	57.5±2.16	0.364±0.39
PEI@IONS	185.5±2.35	0.18±0.006	DNA/PEI@IONS	120.66±3.50	0.293±0.008	127.13±1.18	0.181±0.0042	143.8±0.954	0.205±0.034
PEI@ITOH	132.9±2.85	0.19±0.020	DNA/PEI@ITOH	67.03±9.001	0.914±0.045	78.66±2.42	0.606±0.065	110.37±5.65	0.60±0.01

Table S3. Zeta potential values after DNA coating on PEI@IONPs (DNA/PEI@IONPs) (n=3; mean±SD).

**Zeta Potential (mV)**	**DNA/PEI@IONR**	**DNA/PEI@IONS**	**DNA/PEI@ITOH**
Sample 1	0.18	-0.76	1.05
Sample 2	1.1	-0.91	0.21
Sample 3	1.14	0.14	1.12
Mean±SD	0.90±0.64	-0.51±0.57	0.79±0.51

Western original images

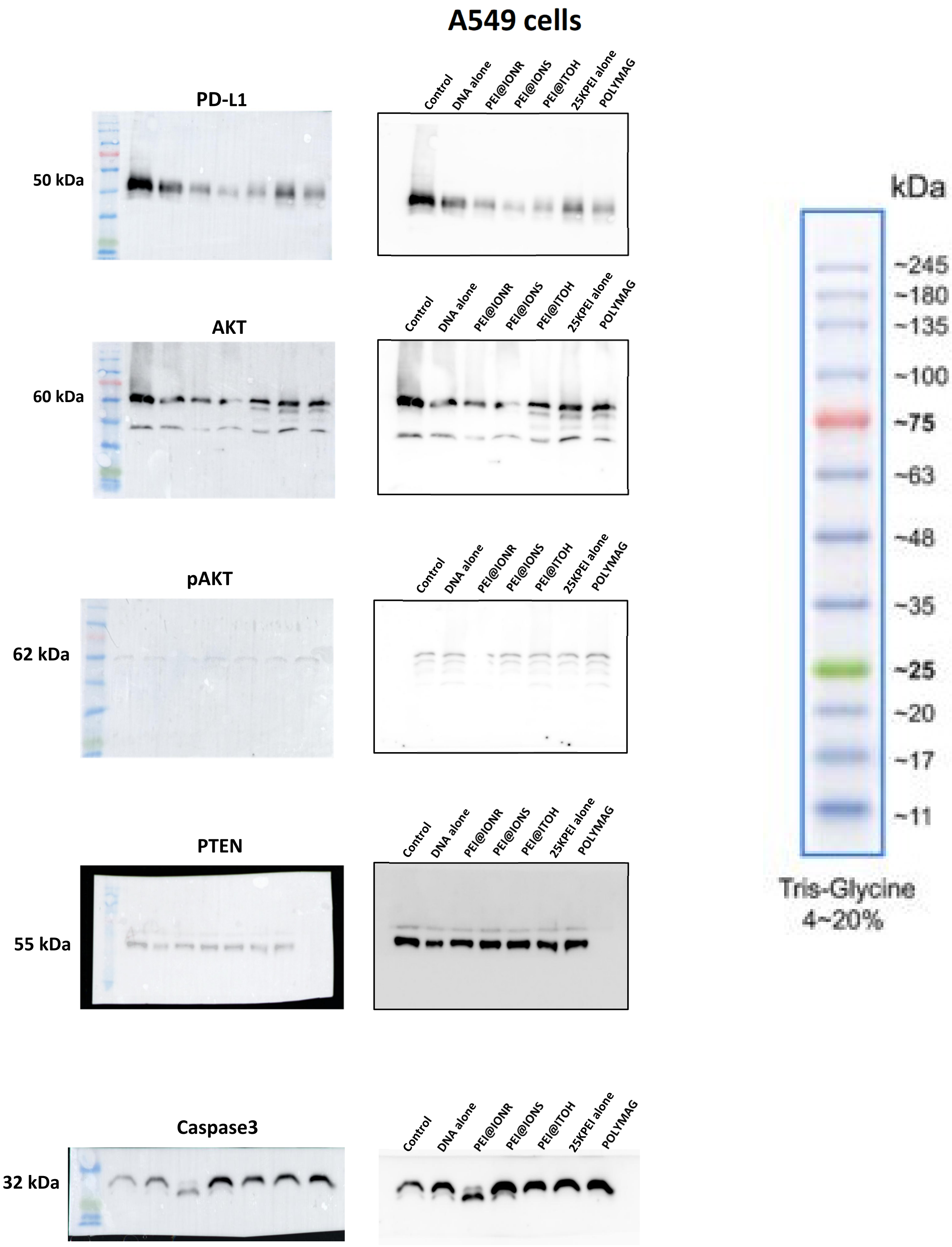

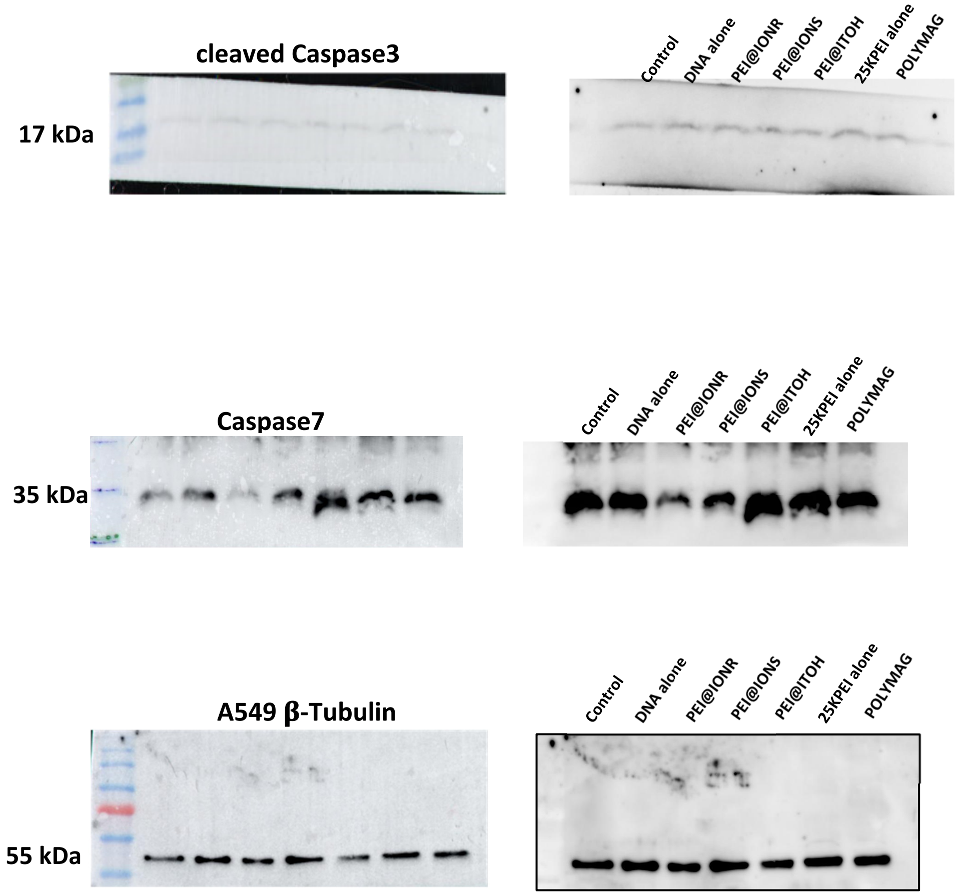
cleaved Caspase3
17 kDa
Control
DNA alone
PEI@IONR
PEI@IONS
PEI@ITOH
25KPEI alone
POLYMAG
Caspase7
35 kDa
Control
DNA alone
PEI@IONR
PEI@IONS
PEI@ITOH
25KPEI alone
POLYMAG
A549 β-Tubulin
55 kDa
Control
DNA alone
PEI@IONR
PEI@IONS
PEI@ITOH
25KPEI alone
POLYMAG

MDA-MB-231 cells

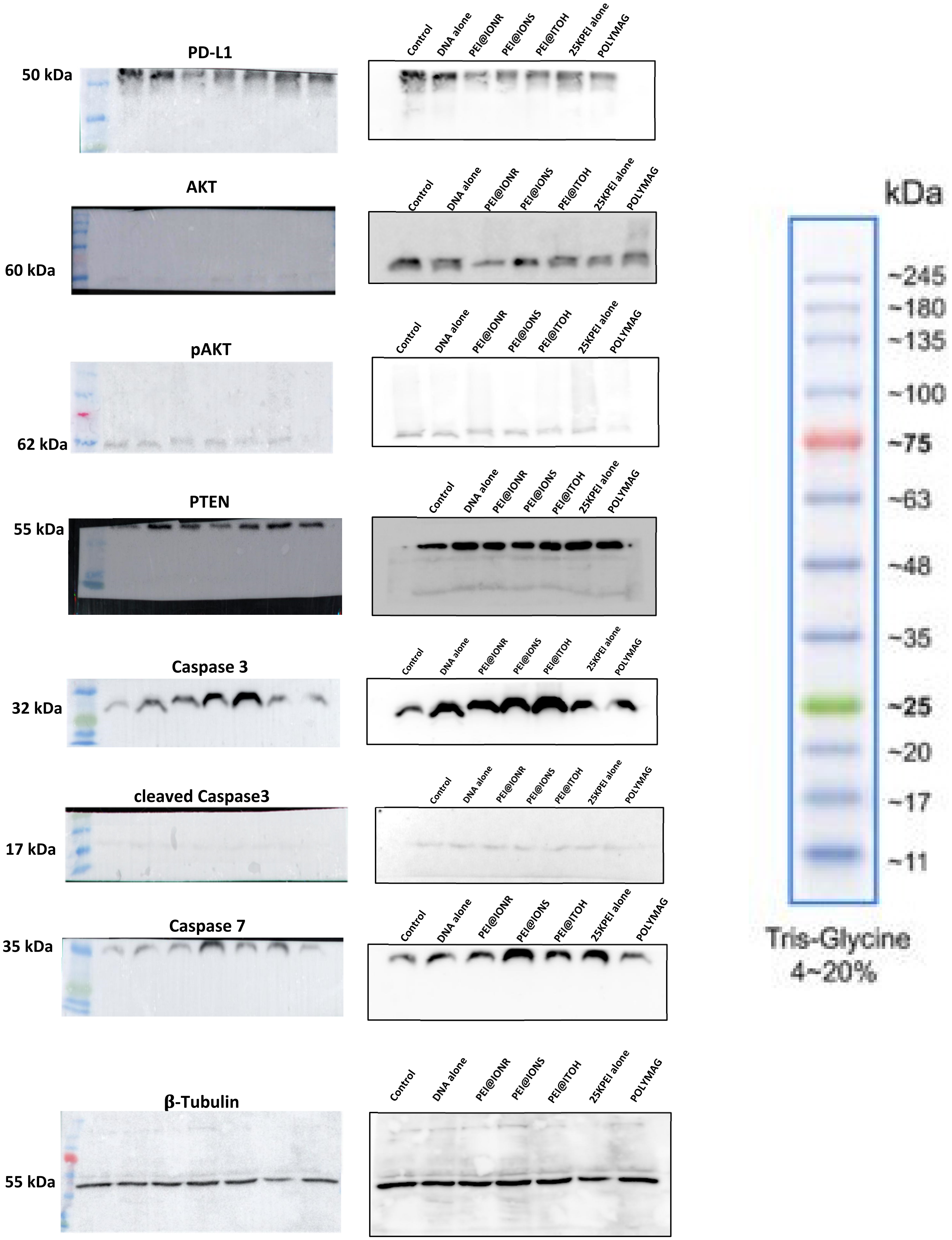

Supplement: Supplementary file 1 [file pharmaceutics-15-00215-s001.zip › pharmaceutics-2104333-supplementary.pdf]
